# Hypoglossal Nerve Stimulation for Obstructive Sleep Apnea: A Systematic Review and Meta-Analysis on Responder-Based Outcomes and Between-Study Heterogeneity

**DOI:** 10.3390/jcm15135180

**Published:** 2026-07-02

**Authors:** Clemens Heiser, Marcel Braun, Colin Huntley, Michael Hutz, Thomas Michael Kaffenberger, Maurits Boon

**Affiliations:** 1 Department of Otorhinolaryngology, Klinikum Rechts Der ISAR, Technical University Munich, 81675 Munich, Germany; 2 ENT Center Mangfall-Inn, 83043 Bad Aibling, Germany; 3 Faculty of Medicine, Translational Neurosciences, University of Antwerp, B-2610 Antwerp, Belgium; 4 Center for Sleep & Telemedicine, Faculty of Medicine, University Duisburg-Essen, 45239 Essen, Germany; 5 Department of Otolaryngology-Head and Neck Surgery, Thomas Jefferson University, Philadelphia, PA 19107, USA; 6 Department of Otolaryngology-Head and Neck Surgery, Section of Sleep Surgery, Rush University Medical Center, Chicago, IL 60612, USA; 7 Department of Otolaryngology-Head & Neck Surgery, University of Pittsburgh Medical Center, University of Pittsburgh, Pittsburgh, PA 15219, USA

**Keywords:** Hypoglossal nerve stimulation, obstructive sleep apnea, Sher response rate

## Abstract

**Background:** Hypoglossal nerve stimulation (HNS) is an established surgical therapy for adults with moderate-to-severe obstructive sleep apnea (OSA) who are intolerant to positive airway pressure. Although aggregate response rates of ~70–80% have been reported, substantial variability across clinical settings remains poorly understood. Prior meta-analyses have largely emphasized pooled continuous outcomes, limiting interpretation of responder-based endpoints and drivers of between-study heterogeneity. **Methods:** A PRISMA-compliant systematic review and meta-analysis was performed. MEDLINE, Embase, and Cochrane CENTRAL were searched from inception through 31 December 2025. Eligible studies enrolled adults with OSA treated with implantable HNS, reported Sher-defined response (≥50% AHI reduction and residual AHI < 20 events/hour), and/or continuous outcomes, and included ≥20 patients. Random-effects models (REML) were applied. Heterogeneity was quantified using I^2^ and τ^2^, with prediction intervals. Meta-regression assessed baseline AHI, BMI, and follow-up duration. Subgroup analyses examined device laterality, stimulation modality, sleep assessment method, and follow-up. **Results:** Thirty-eight studies (39 cohorts; *n* = 3220) were included. The pooled Sher response rate was 74.0% (95% CI 67.6–79.5%). Heterogeneity was substantial. HNS significantly improved all continuous outcomes (AHI −23.3 events/hour; ESS −4.5 points; ODI −14.5 events/hour). Comparative analyses favored HNS over surgical comparators, inactive stimulation, and delayed treatment. Revision and explantation rates were 5% and 4%, respectively. Meta-regression showed no significant effects of baseline AHI, BMI, or follow-up, explaining negligible variance. Subgroups suggested numerically higher response with breathing-synchronized stimulation, but heterogeneity remained high. **Conclusions:** HNS achieves Sher response in approximately three-quarters of appropriately selected CPAP-intolerant OSA patients, with durable clinical benefits and a favorable safety profile. Persistent unexplained heterogeneity highlights limitations of conventional predictors and underscores the need for more granular response determinants.

## 1. Introduction

Obstructive sleep apnea (OSA) is a highly prevalent disorder associated with substantial cardiovascular, metabolic, and neurocognitive morbidity [1,2]. Positive airway pressure (PAP) remains the first-line therapy; however, long-term adherence is limited, leaving a significant proportion of patients undertreated and prompting the development of alternative therapies [3]. Hypoglossal nerve stimulation (HNS) has emerged as an established surgical treatment for adults with moderate-to-severe OSA who are intolerant to PAP, targeting upper airway collapsibility through selective stimulation of the hypoglossal nerve to restore airway patency during sleep.

A historic and still commonly reported metric of treatment success within sleep surgery is the Sher criteria, defined as a ≥50% reduction in apnea–hypopnea index (AHI) with residual AHI < 20 events/hour [4]. Across clinical trials, registries, and prior systematic reviews, Sher response rates following HNS are consistently reported in the range of approximately 70–80% at 6–12 months, with durable effects observed up to five years after implantation [5,6,7,8]. Importantly, heterogeneity in reported outcomes reflects not only differences in patient selection and device characteristics, but also variability in endpoint assessment methodology. While some studies derive outcomes from in-laboratory titration polysomnography (PSG), others utilize full-night fixed-setting PSG or home sleep testing (HST). These approaches may yield systematically different estimates of residual AHI and, consequently, Sher response, and have increasingly been recognized as a relevant contributor to variability in reported effectiveness. These findings have established HNS as an effective therapy in appropriately selected patients.

Despite this apparent consistency at the aggregate level, a closer examination of the literature reveals substantial variability in reported response rates across studies and clinical settings. Real-world cohorts often demonstrate lower success rates compared with controlled trials, and reported outcomes vary depending on patient selection, device generation, follow-up methodology, and definitions of treatment success [9,10]. Furthermore, while prior meta-analyses have primarily focused on pooled improvements in continuous outcomes such as AHI or Epworth Sleepiness Scale (ESS) over time, they have provided limited insight into the clinical interpretability of responder-based outcomes and the extent to which heterogeneity in Sher response can be explained by study-level factors.

At the same time, the therapeutic landscape of HNS is evolving. In addition to established breathing-synchronized unilateral HNS therapy, continuous unilateral HNS and bilateral stimulation therapy have been introduced, with early studies suggesting response rates within the range observed for conventional devices [11,12,13]. However, these data have not yet been systematically integrated into the broader evidence base, and their implications for clinical effectiveness remain unclear.

Taken together, these considerations highlight a critical gap in the current literature: while the average effectiveness of HNS is well established, there is limited understanding of how to interpret Sher response rates across heterogeneous study populations and evolving therapy concepts, and whether commonly reported patient and study characteristics adequately explain variability in outcomes.

Therefore, the aim of the present systematic review and meta-analysis was to provide a contemporary, clinically oriented synthesis of HNS in OSA, with a primary focus on Sher-defined treatment response. Specifically, we sought to (1) quantify Sher’s response across studies, (2) evaluate heterogeneity and its potential drivers using meta-regression and subgroup analyses, (3) distinguish between single-arm and comparative evidence, and (4) incorporate emerging data on continuous unilateral and bilateral HNS therapy within a unified analytical framework.

## 2. Materials and Methods

**Study design and reporting**

This systematic review and meta-analysis were conducted in accordance with the Preferred Reporting Items for Systematic Reviews and Meta-Analyses (PRISMA) guidelines. The analysis followed a predefined framework focusing on responder-based and continuous effectiveness outcomes of HNS in adult OSA.

**Literature search and study selection**

A systematic literature search was performed in MEDLINE (PubMed), Embase, and the Cochrane CENTRAL database from inception to 31 December 2025. Eligible study designs included randomized controlled trials, non-randomized comparative studies, prospective and retrospective cohorts, registry studies, and case series. Study selection was conducted using Covidence by two independent reviewers at the title/abstract and full-text level, with discrepancies resolved by a third reviewer.

**Eligibility criteria**

Eligibility criteria were operationalized using a Population-Intervention-Comparator-Outcome-Technology-Study methodology (PICOTS) framework (Table S1) with the research question defined as follows: In adults with obstructive sleep apnea, what is the effectiveness and safety of implantable hypoglossal nerve stimulation, compared with baseline or alternative therapies, in improving polysomnographic outcomes, patient-reported symptoms, quality of life, and adverse event profiles across different hypoglossal nerve stimulation technologies (including unilateral and bilateral systems and different stimulation modalities), as reported in clinical trials and observational studies?

Studies were included if they enrolled adults (≥18 years) with OSA treated with implantable HNS, reported Sher response and changes in AHI, included ≥20 patients, and used clinical study designs (randomized controlled trials, comparative or observational cohorts, registries).

Both single-arm and comparative studies were eligible. Studies were excluded if they were case reports, technical/bench studies, reviews, or overlapping cohorts with less complete datasets.

**Data extraction and harmonization**

Data extraction included study characteristics, patient demographics (age, Body Mass Index (BMI), sex), baseline disease severity, device characteristics (including laterality and stimulation modality), and outcomes. Risk-of-bias assessment was conducted in Covidence software using a structured domain-based framework informed by ROBINS-I for non-randomized and single-arm observational cohorts (the predominant study design in this evidence base) and RoB 2 for the two included randomized controlled trials. Because ROBINS-I is primarily designed for comparative non-randomized studies, the domain structure was adapted for the single-arm pre-post designs that characterize most HNS cohorts; the six domains applied are listed in the Table S2 header. Sher response rates were analyzed on a per-protocol basis using the number of patients with available follow-up outcome data as the denominator, as most included studies reported outcomes only for these patients, and this approach was considered appropriate to estimate treatment effectiveness in observational HNS cohorts. Where necessary, summary statistics were harmonized using established methods, including the Wan–Luo approach for estimating means and standard deviations from medians and interquartile ranges [14].

For comparative studies, arm-level data were reconstructed and paired at the study level, including classification of comparator types (e.g., surgical, delayed treatment, active HNS comparators). For single-arm studies, pre–post datasets were constructed for effect size estimation. Event counts for Sher response and safety outcomes were derived from reported proportions when not directly available.

**Outcomes**

The primary outcome was Sher response, defined as ≥50% reduction in AHI and residual AHI < 20 events/hour. Secondary outcomes included change in AHI, Oxygen Desaturation Index (ODI), and ESS, revision and explantation rates, and exploratory modifiers including BMI, baseline AHI, follow-up duration, device type, and measurement modality.

**Statistical analysis**

Single-arm and comparative studies were analyzed separately using random-effects meta-analysis with restricted maximum likelihood (REML) estimation.

For single-arm studies, pooled mean changes from baseline were calculated using change-score methods. Variances were derived assuming a pre–post correlation of r = 0.5, with sensitivity analyses for r = 0.3 and r = 0.7. For comparative studies, mean differences were calculated for both end-of-study values and change scores. The Sher response and safety outcomes were pooled using logit-transformed random-effects proportion meta-analysis. For comparative studies, treatment effects were expressed as risk ratios with continuity correction where necessary. Between-study heterogeneity was quantified using τ^2^ and I^2^ statistics. Prediction intervals were calculated to estimate the expected range of effects in future settings. Subgroup analyses were conducted by follow-up duration, device type and laterality, stimulation modality, sleep measurement modality, and study design. For proportion outcomes, pooled estimates were derived using a random-effects meta-analysis of study-level response rates rather than crude aggregation of responders across studies, thereby appropriately accounting for both within-study variance and substantial between-study heterogeneity.

Meta-regression analyses evaluated potential moderators, including baseline AHI, BMI, follow-up duration, and device type, with multivariable models applied where data permitted. Sensitivity analyses included leave-one-out influence analyses, variation in pre–post correlation assumptions, and restriction to comparator subgroups in comparative analyses. Funnel plots and Egger’s regression tests were used to assess small-study effects when ≥10 studies were available.

To further explore potential sources of heterogeneity related to endpoint ascertainment, post hoc meta-regression analyses were performed, evaluating the sleep-study assessment context. Specifically, models assessed (1) HNS sleep-setting context (titration vs. fixed-setting), (2) combined assessment context (sleep-study modality and setting), and (3) outcome assessment modality alone. These analyses aimed to quantify the contribution of measurement context to between-study heterogeneity.

All analyses were conducted in R (R Foundation for Statistical Computing) using R Studio (Version 2025.05.0+496) and the *meta* and *metafor* packages.

## 3. Results

**Study selection and characteristics**

**Figure 1:**
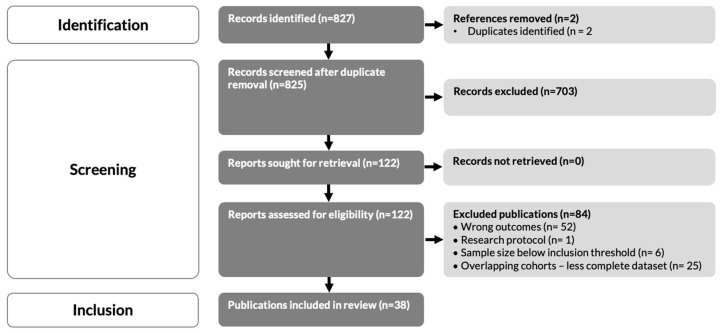
PRISMA flow chart on literature identification and selection.

A total of 38 studies comprising 39 cohorts and 3220 patients were included in the quantitative synthesis (Figure 1). The evidence base was predominantly observational, with most studies conducted in single-center settings and using unilateral, breathing-synchronized HNS therapy (Table 3A,B). Follow-up ranged from 3 to 60 months, with the majority of cohorts reporting outcomes within 12 months (weighted mean follow-up = 10.2 ± 9.4 months). Overall, risk of bias was predominantly moderate to high across included studies, reflecting the observational nature of the evidence base, with most studies limited by lack of randomization, potential selection bias, and incomplete reporting of key methodological domains (Table S2).

Polysomnography (PSG) was the primary outcome assessment modality in 60% of cohorts, while 12.5% used home sleep testing (HST) alone and 27.5% used mixed modalities. Among PSG-based cohorts, reporting of assessment context varied, with both titration-based and fixed-setting full-night protocols represented. Total implanted sample sizes are reported descriptively, whereas pooled Sher response estimates are based on patients with available follow-up assessments.

**Primary outcome: Sher response**

**Figure 2:**
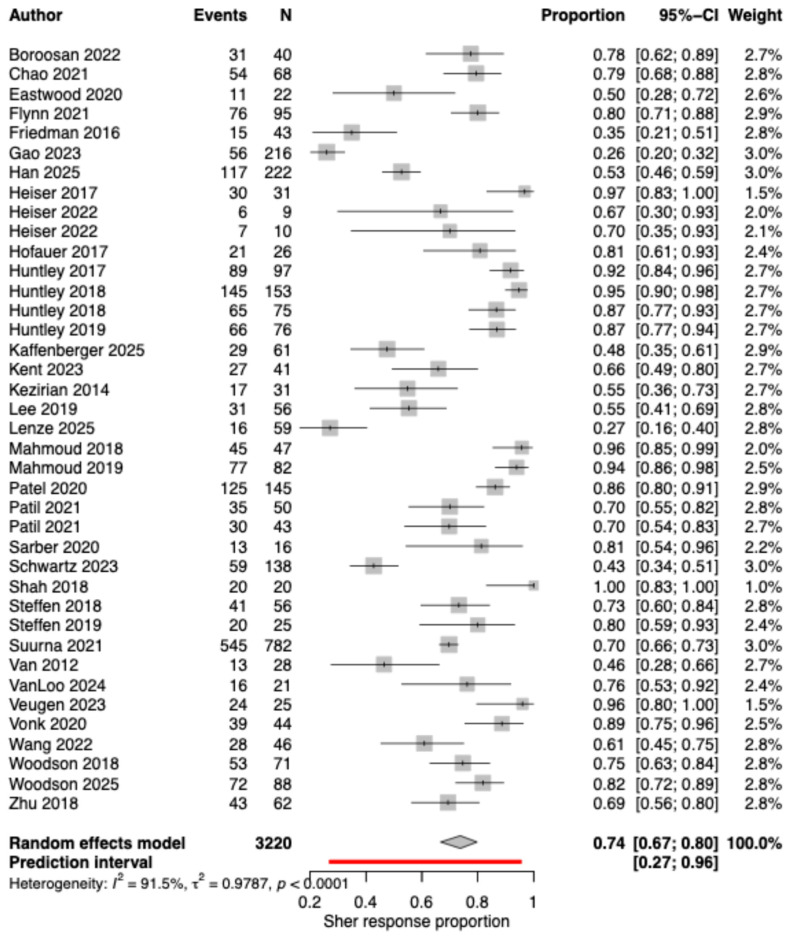
Forest plot of primary outcome Sher response.

Across 39 cohorts (n = 3220), the pooled Sher response rate was 74.0% (95% CI 67.6–79.5%, Figure 2). This corresponds to approximately three out of four patients achieving a clinically meaningful response following HNS therapy. These estimates reflect pooled results across studies using both titration-based and fixed-setting assessments and should therefore be interpreted in the context of differing outcome ascertainment methodologies. Between-study heterogeneity was substantial (I^2^ ≈ 91%), with a wide prediction interval (27% to 96%), indicating marked variability in response rates across clinical settings. Individual cohort estimates ranged from approximately 26% to 100%. Post hoc meta-regression analyses demonstrated that sleep-study assessment context was a statistically significant moderator of Sher response. In the primary model evaluating HNS sleep-setting context, titration-based assessments were associated with significantly higher response estimates compared with fixed-setting contexts (QM(df = 2) = 9.12, *p* = 0.010; R^2^ = 21.0%). A combined assessment-context model incorporating both sleep-study modality and setting yielded consistent findings (QM(df = 3) = 8.60, *p* = 0.035; R^2^ = 16.9%), with all non-titration contexts (fixed PSG, fixed HST, and mixed/unclear) showing significantly lower response estimates relative to the reference.

Across all models, residual heterogeneity remained substantial (I^2^ ≈ 89–91%, QE *p* < 0.001), indicating that while assessment context explains a meaningful proportion of variability, the majority of between-study heterogeneity remains unexplained.

**Continuous outcomes**

Across all continuous outcomes, HNS was associated with a significant improvement in subjective and objective OSA severity (Table 1, Figures S1–S3). The meta-analysis revealed a reduction in AHI, with a pooled mean change of −23.3 events/hour (95% CI −25.6 to −21.1; *p* < 0.001). Heterogeneity was high (I^2^ ≈ 96.8%), with a prediction interval of −37.0 to −9.7 events/hour. Daytime sleepiness, measured with ESS, improved significantly, with a pooled mean change of −4.5 points (95% CI −5.0 to −4.1; *p* < 0.001). Heterogeneity remained substantial (I^2^ ≈ 86%), although the prediction interval remained entirely below zero. Nightly oxygen desaturations, measured with the ODI, were reduced by 14.5 events/hour (95% CI −16.8 to −12.2; *p* < 0.001). Heterogeneity was high (I^2^ ≈ 83.7%), with a consistent direction of effect across studies.

**Safety outcomes**

Across the available safety cohorts, hypoglossal nerve stimulation demonstrated a favorable device-related safety profile (Figures S4 and S5). The pooled revision rate was 5% (95% CI 3–7%) across nine cohorts (514 patients), with low between-study heterogeneity, indicating consistent procedural safety across centers. Similarly, the pooled explantation rate was 4% (95% CI 2–9%) across 10 cohorts (417 patients), suggesting that permanent device removal was uncommon and long-term device retention was generally high. Leave-one-out sensitivity analyses confirmed the robustness of both safety endpoints, with revision and explantation estimates remaining stable across all iterations.

**Comparative effectiveness**

**Figure 3:**
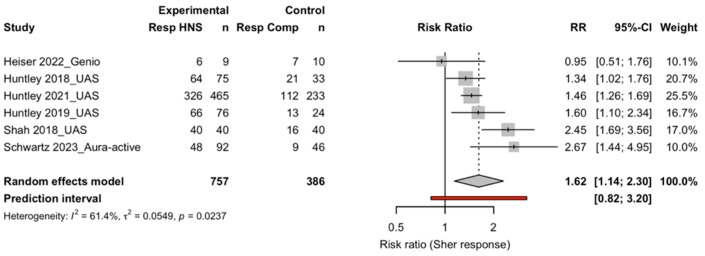
Forest plot of Sher response rate across studies with comparator.

Six comparative studies evaluated HNS against surgical comparators, inactive stimulation controls, or delayed treatment strategies (Figure 3). HNS was associated with a significantly higher likelihood of achieving Sher response (RR 1.62; 95% CI 1.14–2.30). Heterogeneity was moderate (I^2^ ≈ 61%). A subgroup restricted to surgical comparators showed a similar effect direction but did not reach statistical significance.

**Heterogeneity and moderator analyses**

Substantial between-study heterogeneity was observed for the primary outcome of Sher response (I^2^ ≈ 91%), with a wide prediction interval (27% to 96%) indicating considerable variability in treatment response across clinical settings. This degree of heterogeneity was consistently observed across analyses and was not materially reduced by subgroup stratification.

**Figure 4:**
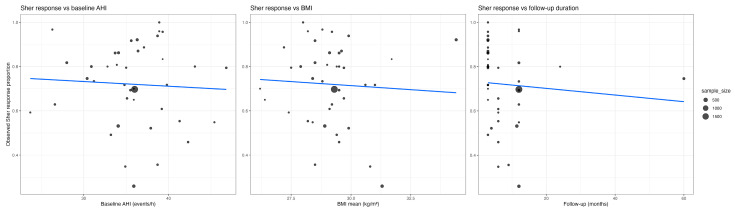
Meta-regression of Sher response vs. baseline AHI, BMI and follow-up duration.

Meta-regression analyses were conducted to evaluate potential moderators of Sher response, including baseline AHI, BMI, and follow-up duration (Table 2 and Figure 4). None of these variables was a statistically significant predictor of treatment response, and the proportion of explained variance was negligible. These findings were consistent across univariable and multivariable models, indicating that commonly reported study-level characteristics do not explain the observed variability in outcomes.

Subgroup and exploratory stratified analyses provided additional descriptive insights but did not meaningfully reduce heterogeneity. Stratification by follow-up duration showed numerically stable Sher response rates across time points, with no clear trend toward attenuation or improvement over longer follow-up periods, consistent with the non-significant meta-regression for follow-up duration. Similarly, the predominance of single-arm observational cohorts, together with the limited number of comparative studies, suggests that study design alone is unlikely to explain the observed variability in response rates. This was particularly evident in the exploratory sleep-modality subgroup, in which PSG-based cohorts showed pooled Sher response rates of approximately 79%, compared with 73% for HST-based cohorts and 59% for mixed-assessment studies, while substantial heterogeneity persisted within all subgroups. These findings suggest that differences in sleep-study methodology, particularly the distinction between titration-based and fixed-setting assessments, represent a more relevant source of variability than sleep modality alone.

Subgroup analysis by stimulation modality demonstrated numerically higher Sher response rates for breathing-synchronized systems (76%) and phasic/bilateral systems (69%), compared with continuous stimulation approaches (41%). These differences are directionally consistent with physiological expectations but should be interpreted with caution, as the number of studies in certain subgroups, particularly continuous stimulation, was limited. Importantly, heterogeneity remained high within all subgroups.

Across all analyses, no single factor or subgroup explained the observed variability in treatment response. These findings indicate that heterogeneity is likely multifactorial and reflects a combination of clinical, methodological, and center-level differences that are not captured by standard study-level variables.

**Sensitivity analyses and publication bias**

Leave-one-out analyses demonstrated stable pooled estimates across all outcomes, indicating that the observed treatment effects were not driven by individual studies. Likewise, correlation-based sensitivity analyses showed only minimal variation in the pooled continuous outcome estimates across assumed pre–post correlations, confirming the robustness of the AHI, ESS, ODI and safety findings to variance assumptions (Figures S6–S11). In contrast, Egger’s regression test indicated significant funnel plot asymmetry for Sher response (z = 3.97, *p* < 0.001), suggesting the possibility of small-study effects or selective dissemination affecting the primary responder endpoint. However, given the substantial clinical and methodological heterogeneity across cohorts, including variation in patient selection, sleep-study modality, and center experience, this finding should be interpreted cautiously, particularly in the context of heterogeneous study designs and evidence that outcome assessment methodology influences observed treatment response. For the continuous outcomes, evidence for funnel plot asymmetry was less consistent, with no clear statistical signal for AHI or ODI and only borderline evidence for ESS.

## 4. Discussion

This systematic review and meta-analysis demonstrate that HNS is an effective therapy for OSA in a population that has typically failed conventional treatment options. Across a large and heterogeneous evidence base, approximately three out of four patients achieved Sher-defined treatment response, accompanied by substantial improvements in AHI, daytime sleepiness, and oxygen desaturation. These findings are consistent with prior systematic reviews and long-term follow-up studies, which have established HNS as a clinically meaningful and durable second-line therapy for CPAP-intolerant patients [5,6,7,8,15,16]. Importantly, this level of effectiveness is observed in a population with limited alternatives, reinforcing the clinical value of HNS within the current OSA treatment landscape.

This study highlights that the endpoint assessment context represents a statistically significant contributor to between-study variability. Post hoc meta-regression analyses demonstrated that titration-based sleep studies were associated with higher reported Sher response compared with fixed-setting or non-titration assessments, with measurement context explaining approximately 17–21% of between-study heterogeneity. These findings were consistent across multiple model specifications and indicate that part of the observed variability reflects differences in outcome ascertainment rather than underlying biological variation alone.

At the same time, the present analysis shows that Sher response rates vary substantially across studies, with wide prediction intervals indicating that outcomes are not uniform across clinical settings. Rather than weakening the overall effectiveness signal, this variability should be interpreted as reflecting the real-world complexity of OSA and its treatment, where outcomes depend on patient phenotype, anatomical factors, treatment execution, and differences in endpoint ascertainment methodology across studies. In this context, Sher response is best understood not as a fixed performance metric, but as a context-dependent outcome whose interpretation requires consideration of clinical and methodological factors. This perspective extends prior meta-analyses, which have primarily focused on average treatment effects or temporal durability, by highlighting the variability underlying these averages.

A central finding of this study is that commonly reported study-level variables, including baseline AHI, body mass index (BMI), and follow-up duration, did not explain the observed heterogeneity in treatment response. While these factors are often used in clinical decision-making and trial design, the present results suggest that they do not adequately capture the determinants of HNS effectiveness. Instead, treatment response is likely driven by more specific and clinically relevant factors, including upper airway anatomy and physiology, collapse patterns on drug-induced sleep endoscopy, and the quality of implantation and titration [17,18,19,20]. These elements are fundamental to ENT practice but are not consistently reported in the literature, limiting their integration into meta-analytic models. The findings therefore support a shift from reliance on simplified baseline characteristics toward more refined phenotyping and standardized reporting.

The present findings further demonstrate that differences in outcome measurement contribute meaningfully to variability in reported effectiveness. While prior work has highlighted differences between polysomnography and home sleep testing, the current analysis extends this by showing that the distinction between titration-based and fixed-setting assessments is particularly relevant [20,21]. Titration studies, which involve active adjustment of stimulation parameters during assessment, were associated with higher reported response rates compared with full-night fixed-setting PSG or HST. Importantly, although this factor explained a measurable proportion of heterogeneity, substantial residual variability remained, indicating that measurement context is one of several contributors rather than a dominant determinant of treatment response.

An important strength of this analysis is the inclusion of comparative evidence, which remains limited in the HNS literature. The finding that HNS is associated with a higher likelihood of achieving Sher response compared to alternative or delayed treatment strategies supports the interpretation that the observed benefits are not solely attributable to pre–post study designs. This is consistent with prior controlled analyses demonstrating non-inferiority or superiority of HNS compared to positive airway pressure in selected populations [22,23] and strengthens the overall evidence base for HNS as an effective therapeutic option.

The inclusion of emerging bilateral stimulation therapy provides additional context for interpreting these findings. Early evidence suggests that bilateral hypoglossal nerve stimulation achieves outcomes that are directionally comparable to established unilateral systems, without a clear signal of reduced effectiveness [11,12,24]. While the available data remain limited and do not permit formal comparative conclusions, these observations are clinically reassuring and suggest that effectiveness may be preserved across different stimulation concepts. Importantly, this further supports the interpretation that treatment outcomes are driven less by stimulation application alone and more by patient selection and clinical implementation.

Safety outcomes were favorable, with low rates of revision and explantation, consistent with previous systematic reviews [25,26]. Together with the observed effectiveness, this supports a favorable benefit–risk profile for HNS in appropriately selected patients. However, as highlighted in prior work, variability in adverse event reporting remains a limitation, and more standardized frameworks would improve comparability across studies (Table 3A,B).

Several limitations should be considered. First, the evidence base is predominantly observational, with limited randomized data, which introduces potential bias related to patient selection and confounding. Second, substantial heterogeneity was observed across studies, reflecting both clinical and methodological differences that could not be fully accounted for. Although post hoc analyses identified assessment context as a statistically significant moderator, this factor explained only a portion of the observed heterogeneity, and residual variability remained substantial. Third, key determinants of treatment response, particularly anatomical characteristics and titration protocols, were inconsistently reported and could not be incorporated into the analyses. Fourth, subgroup analyses, including those by stimulation modality, were exploratory and based on limited data in certain categories. Fifth, the use of Sher response as the primary endpoint, while clinically intuitive and widely adopted, represents a dichotomization of a continuous outcome and may not fully capture the spectrum of clinically meaningful improvement. Finally, Sher response rates were analyzed on a per-protocol basis, using patients with available follow-up data as the denominator. This approach was necessitated by the fact that most included studies reported outcomes only for assessed patients and did not consistently provide total implanted sample sizes sufficient to reconstruct an intention-to-treat denominator across all cohorts. Consequently, the pooled estimate is susceptible to attrition bias: patients lost to follow-up may be systematically more likely to be non-responders, and the reported 74.0% response rate may therefore overestimate the true population-level value. This concern is most pronounced in cohorts with substantial attrition. A uniform worst-case sensitivity analysis could not be applied across all cohorts due to inconsistent reporting of total implanted denominators. Future meta-analyses in this field should standardize on intention-to-treat denominators to enable this analysis routinely.

## 5. Conclusions

HNS is an effective and clinically valuable therapy for patients with OSA who are intolerant to conventional treatment, while demonstrating that treatment response varies substantially across clinical settings. These findings highlight the importance of patient selection, outcome assessment, and clinical expertise in determining treatment success, and suggest that future advances in the field will depend on improved phenotyping and standardization rather than further refinement of average efficacy estimates.

## Figures and Tables

**Table 1:** Pooled effect estimates for continuous outcomes (AHI, ODI, ESS).

Outcome	Pooled Estimate (95% CI Lower Bound, 95% CI Upper Bound)	Prediction Interval (Lower Bound, Upper Bound)	I^2^	Tau^2^
AHI	−23.3 (−25.7, −21.1)	−37.2, −9.6	96.8	48.4
ODI	−14.5 (−16.7, −12.1)	−23.3, −5.5	84.2	19.3
ESS	−4.5 (−5.0, −4.1)	−6.7, −2.3	86.4	1.2

**Table 2:** Meta-regression analyses evaluating the association between Sher response and selected study-level variables.

Moderator	Effect Estimate (β)	95% CI	*p*-Value
Baseline AHI	−0.011	−0.082 to 0.060	0.761
BMI	−0.025	−0.215 to 0.264	0.840
Follow-up (months)	−0.009	−0.044 to 0.025	0.602

**Table 3:** (**A**) List of included cohorts (pre-post analysis). (**B**) List of included cohorts (comparative analysis).

**(A)**							
**Cohort**	**Title**	**Study Design**	**Study Type**	**Device**	**Laterality**	**Stimulation Modality**	**Implant Configuration**
Boroosan 2022 [27]	Clinical Predictors of OSA Treatment Success Following Implantation of a Hypoglossal Nerve Stimulation Device.	Retrospective cohort study	Single-center	Inspire UAS (Inspire Medical Systems, Golden Valley, MN, USA)	Unilateral	Breathing-synchronized	Fully implantable
Chao 2020 [28]	Predictors of success in hypoglossal nerve stimulator implantation for obstructive sleep apnea.	Retrospective cohort study	Single-center	Inspire UAS	Unilateral	Breathing-synchronized	Fully implantable
Eastwood 2020 [12]	Bilateral hypoglossal nerve stimulation for treatment of adult obstructive sleep apnoea.	Prospective cohort study	Multi-center	Nyxoah Genio (Nyxoah SA, Mont-Saint-Guibert, Belgium)	Bilateral	Phasic	Partial implantable
Flynn 2021 [29]	The Effect of Lateral Pharyngeal Collapse Patterns on Therapy Response in Upper Airway Stimulation Surgery.	Retrospective cohort study	Single-center	Inspire UAS	Unilateral	Breathing-synchronized	Fully implantable
Friedman 2016 [30]	Targeted hypoglossal nerve stimulation for the treatment of obstructive sleep apnea: Six-month results.	Prospective cohort study	Multi-center	LivaNova aura6000 (LivaNova, London, United Kingdom)	Unilateral	Continuous	Fully implantable
Gao 2023 [31]	Hypoglossal Nerve Stimulation Therapy Outcomes in Apnea—Versus Hypopnea-Predominant Patients.	Retrospective cohort study	Single-center	Inspire UAS	Unilateral	Breathing-synchronized	Fully implantable
Han 2025 [32]	Impact of Postoperative Weight Changes on Hypoglossal Nerve Stimulation Success for Obstructive Sleep Apnea.	Retrospective cohort study	Single-center	Inspire UAS	Unilateral	Breathing-synchronized	Fully implantable
Heiser 2017 [33]	Selective upper airway stimulation for obstructive sleep apnea: a single center clinical experience.	Prospective cohort study	Single-center	Inspire UAS	Unilateral	Breathing-synchronized	Fully implantable
Heiser 2022_Genio [24]	Bilateral vs. Unilateral Hypoglossal Nerve Stimulation in Patients with Obstructive Sleep Apnea.	Prospective cohort study	Single-center	Nyxoah Genio	Bilateral	Phasic	Partially implantable
Heiser 2022_UAS [24]	Bilateral vs. Unilateral Hypoglossal Nerve Stimulation in Patients with Obstructive Sleep Apnea.	Prospective cohort study	Single-center	Inspire UAS	Unilateral	Breathing-synchronized	Fully implantable
Hofauer 2017 [34]	Effects of upper airway stimulation on sleep architecture in patients with obstructive sleep apnea.	Retrospective cohort study	Single-center	Inspire UAS	Unilateral	Breathing-synchronized	Fully implantable
Huntley 2017 [35]	Upper Airway Stimulation for Treatment of Obstructive Sleep Apnea: An Evaluation and Comparison of Outcomes at Two Academic Centers.	Retrospective cohort study	Multi-center	Inspire UAS	Unilateral	Breathing-synchronized	Fully implantable
Huntley 2018_study1 [36]	Upper Airway Stimulation in Patients with Obstructive Sleep Apnea and an Elevated Body Mass Index: A Multi-institutional Review.	Retrospective cohort study	Multi-center	Inspire UAS	Unilateral	Breathing-synchronized	Fully implantable
Huntley 2018_study2 [37]	Comparing Upper Airway Stimulation to Expansion Sphincter Pharyngoplasty: A Single University Experience.	Retrospective cohort study	Single-center	Inspire UAS	Unilateral	Breathing-synchronized	Fully implantable
Huntley 2019 [38]	Comparing Upper Airway Stimulation to Transoral Robotic Base of Tongue Resection for Treatment of Obstructive Sleep Apnea.	Retrospective cohort study	Single-center	Inspire UAS	Unilateral	Breathing-synchronized	Fully implantable
Kaffenberger 2025 [39]	How we measure hypoglossal nerve stimulator outcome matters: titration vs. single amplitude efficacy sleep studies.	Retrospective cohort study	Multi-center	Inspire UAS	Unilateral	Breathing-synchronized	Fully implantable
Kent 2023 [20]	Comparison of clinical pathways for hypoglossal nerve stimulation management: in-laboratory titration polysomnography vs. home-based efficacy sleep testing.	Randomized controlled trial	Multi-center	Inspire UAS	Unilateral	Breathing-synchronized	Fully implantable
Kezirian 2014 [40]	Hypoglossal nerve stimulation improves obstructive sleep apnea: 12-month outcomes.	Prospective cohort study	Multi-center	Apnex HGNS (Apnex Medical, St. Paul, MN, United States)	Unilateral	Breathing-synchronized	Fully implantable
Lee 2019 [41]	Therapeutic Positive Airway Pressure Level Predicts Response to Hypoglossal Nerve Stimulation for Obstructive Sleep Apnea.	Retrospective cohort study	Multi-center	Inspire UAS	Unilateral	Breathing-synchronized	Fully implantable
Lenze 2025 [42]	Evaluating changes in hypoglossal nerve stimulator use over time and long-term adherence.	Retrospective cohort study	Single-center	Inspire UAS	Unilateral	Breathing-synchronized	Fully implantable
Mahmoud 2017 [43]	Upper airway stimulation therapy and prior airway surgery for obstructive sleep apnea.	Retrospective cohort study	Single-center	Inspire UAS	Unilateral	Breathing-synchronized	Fully implantable
Mahmoud 2019 [44]	Outcomes of Hypoglossal Nerve Upper Airway Stimulation among Patients with Isolated Retropalatal Collapse.	Retrospective cohort study	Single-center	Inspire UAS	Unilateral	Breathing-synchronized	Fully implantable
Patel 2020 [45]	Effect of Gender. Age. and Profound Disease on Upper Airway Stimulation Outcomes.	Retrospective cohort study	Single-center	Inspire UAS	Unilateral	Breathing-synchronized	Fully implantable
Patil 2021_study1 [46]	Hypoglossal Nerve Stimulation in Veterans with Comorbid Insomnia and Sleep Apnea.	Retrospective cohort study	Single-center	Inspire UAS	Unilateral	Breathing-synchronized	Fully implantable
Patil 2021_study2 [47]	Hypoglossal Nerve Stimulation: Outcomes in Veterans with Obstructive Sleep Apnea and Common Comorbid Post-traumatic Stress Disorder.	Retrospective cohort study	Single-center	Inspire UAS	Unilateral	Breathing-synchronized	Fully implantable
Sarber 2020 [48]	Hypoglossal Nerve Stimulator Outcomes for Patients Outside the U.S. FDA Recommendations.	Retrospective cohort study	Single-center	Inspire UAS	Unilateral	Breathing-synchronized	Fully implantable
Schwartz 2023 [13]	Targeted Hypoglossal Nerve Stimulation for Patients with Obstructive Sleep Apnea: A Randomized Clinical Trial.	Randomized controlled trial	Multi-center	LivaNova aura6000	Unilateral	Continuous	Fully implantable
Shah 2018 [49]	Uvulopalatopharyngoplasty vs. CN XII stimulation for treatment of obstructive sleep apnea: A single institution experience.	Retrospective cohort study	Single-center	Inspire UAS	Unilateral	Breathing-synchronized	Fully implantable
Steffen 2018 [10]	Outcome after one year of upper airway stimulation for obstructive sleep apnea in a multi-center German post-market study.	Prospective cohort study	Multi-center	Inspire UAS	Unilateral	Breathing-synchronized	Fully implantable
Steffen 2019 [50]	Upper Airway Stimulation Before. After. or Without Uvulopalatopharyngoplasty: A Two-Year Perspective.	Retrospective cohort study	Single-center	Inspire UAS	Unilateral	Breathing-synchronized	Fully implantable
Suurna 2021 [9]	Impact of Body Mass Index and Discomfort on Upper Airway Stimulation: ADHERE Registry 2020 Update.	Retrospective cohort study	Multi-center	Inspire UAS	Unilateral	Breathing-synchronized	Fully implantable
Van de Heyning 2012 [51]	Implanted upper airway stimulation device for obstructive sleep apnea.	Prospective cohort study	Single-center	Inspire UAS	Unilateral	Breathing-synchronized	Fully implantable
Van Loo 2024 [52]	Hypoglossal Nerve Stimulation Therapy in a Belgian Cohort of Obstructive Sleep Apnea Patients.	Prospective cohort study	Single-center	Inspire UAS	Unilateral	Breathing-synchronized	Fully implantable
Veugen 2023 [53]	Upper Airway Stimulation in Patients with Obstructive Sleep Apnea: Long-Term Surgical Success. Respiratory Outcomes. and Patient Experience.	Retrospective cohort study	Single-center	Inspire UAS	Unilateral	Breathing-synchronized	Fully implantable
Vonk 2020 [54]	Short-term results of upper airway stimulation in obstructive sleep apnoea patients: the Amsterdam experience.	Retrospective cohort study	Single-center	Inspire UAS	Unilateral	Breathing-synchronized	Fully implantable
Wang 2022 [55]	Neurophysiological profiles of responders and non-responders to hypoglossal nerve stimulation: a single-institution study.	Retrospective cohort study	Single-center	Inspire UAS	Unilateral	Breathing-synchronized	Fully implantable
Woodson 2018 [6]	Upper Airway Stimulation for Obstructive Sleep Apnea: 5-Year Outcomes.	Prospective cohort study	Multi-center	Inspire UAS	Unilateral	Breathing-synchronized	Fully implantable
Woodson 2025 [11]	Bilateral hypoglossal nerve stimulation for obstructive sleep apnea: a non-randomized clinical trial.	Prospective cohort study	Multi-center	Nyxoah Genio	Bilateral	Phasic	Partially implantable
Zhu 2018 [56]	Selective upper airway stimulation in older patients.	Retrospective cohort study	Single-center	Inspire UAS	Unilateral	Breathing-synchronized	Fully implantable
**(B)**							
**Cohort**	**Title**	**Study Design**	**Study Arm**	**Device**	**Laterality**	**Stimulation Modality**	**Implant Configuration**
Heiser 2022_Genio [24]	Bilateral vs. Unilateral Hypoglossal Nerve Stimulation in Patients with Obstructive Sleep Apnea.	Prospective comparative cohort study	HNS	Nyxoah Genio	Nyxoah Genio	Bilateral	Phasic
Heiser 2022_UAS [24]	Bilateral vs. Unilateral Hypoglossal Nerve Stimulation in Patients with Obstructive Sleep Apnea.	Prospective comparative cohort study	Comparator	Inspire UAS	Inspire UAS	Unilateral	Breathing-synchronized
Huntley 2018_UAS [37]	Comparing Upper Airway Stimulation to Expansion Sphincter Pharyngoplasty: A Single University Experience.	Retrospective matched-cohort study	HNS	Inspire UAS	Inspire UAS	Unilateral	Breathing-synchronized
Huntley 2018_ESP [37]	Comparing Upper Airway Stimulation to Expansion Sphincter Pharyngoplasty: A Single University Experience.	Retrospective matched-cohort study	Comparator	Surgery ESP	n/a	n/a	n/a
Huntley 2019_UAS [38]	Comparing Upper Airway Stimulation to Transoral Robotic Base of Tongue Resection for Treatment of Obstructive Sleep Apnea.	Retrospective matched-cohort study	HNS	Inspire UAS	Inspire UAS	Unilateral	Breathing-synchronized
Huntley 2019_TORS [38]	Comparing Upper Airway Stimulation to Transoral Robotic Base of Tongue Resection for Treatment of Obstructive Sleep Apnea.	Retrospective matched-cohort study	Comparator	Surgery (TORS)	n/a	n/a	n/a
Huntley 2021_UAS [57]	Comparison of Traditional Upper Airway Surgery and Upper Airway Stimulation for Obstructive Sleep Apnea.	Retrospective matched-cohort study	HNS	Inspire UAS	Inspire UAS	Unilateral	Breathing-synchronized
Huntley 2021_UA-Sx [57]	Comparison of Traditional Upper Airway Surgery and Upper Airway Stimulation for Obstructive Sleep Apnea.	Retrospective matched-cohort study	Comparator	Surgery (diverse)	n/a	n/a	n/a
Schwartz 2023_Aura-active [13]	Targeted Hypoglossal Nerve Stimulation for Patients with Obstructive Sleep Apnea: A Randomized Clinical Trial.	Randomized controlled trial	HNS	Active stimulation	LivaNova aura6000	Unilateral	Continuous
Schwartz 2023_Aura-control [13]	Targeted Hypoglossal Nerve Stimulation for Patients with Obstructive Sleep Apnea: A Randomized Clinical Trial.	Randomized controlled trial	Comparator	Control (Stimulation not active)	LivaNova aura6000	Unilateral	Continuous
Shah 2018_UAS [49]	Uvulopalatopharyngoplasty vs. CN XII stimulation for treatment of obstructive sleep apnea: A single institution experience.	Retrospective matched-cohort study	HNS	Inspire UAS	Inspire UAS	Unilateral	Breathing-synchronized
Shah 2018_UPPP [49]	Uvulopalatopharyngoplasty vs. CN XII stimulation for treatment of obstructive sleep apnea: A single institution experience.	Retrospective cohort study	Comparator	Surgery (UPPP)	n/a	n/a	n/a

## Data Availability

Data is available from the corresponding author upon reasonable request.

## Supplementary Materials

The following supporting information can be downloaded at: https://www.mdpi.com/article/10.3390/jcm15135180/s1, Figure S1: Forest plot of AHI changes (pre-post analysis); Figure S2: Forest plot of ESS changes (pre-post analysis); Figure S3: Forest plot of ODI changes (pre-post analysis); Figure S4: Forest plot of revision rates; Figure S5: Forest plot of explantation rates; Figure S6: Eggers funnel plot for outcome Sher response; Figure S7: Eggers funnel plot for outcome AHI change; Figure S8: Eggers funnel plot for outcome ESS change; Figure S9: Eggers funnel plot for outcome ODI change; Figure S10: Eggers funnel plot for outcome Revision rate; Figure S11: Eggers funnel plot for outcome Explantation rate; Table S1: PICOTS question, PICOTS formulation and derived search string; Table S2: Risk of bias assessment.

## Abbreviations

The following abbreviations are used in this manuscript:

AHI	Apnea–Hypopnea Index
BMI	Body Mass Index
CI	Confidence Interval
CPAP	Continuous Positive Airway Pressure
ESS	Epworth Sleepiness Scale
HNS	Hypoglossal Nerve Stimulation
HST	Home Sleep Test
I^2^	I-squared (measure of heterogeneity)
ODI	Oxygen Desaturation Index
OSA	Obstructive Sleep Apnea
PAP	Positive Airway Pressure
PICOTS	Population, Intervention, Comparator, Outcome, Technology, Study design
PRISMA	Preferred Reporting Items for Systematic Reviews and Meta-Analyses
PSG	Polysomnography
QE	Cochran’s Q statistic for heterogeneity
QM	Test statistic for moderators in meta-regression
REML	Restricted Maximum Likelihood
RR	Risk Ratio
**τ^2^**	Between-study variance
