# Supplementary material for: Hypoglossal Nerve Stimulation for Obstructive Sleep Apnea: A Systematic Review and Meta-Analysis on Responder-Based Outcomes and Between-Study Heterogeneity
Source: J Clin Med. 2026 Jul 2;15(13):5180. doi: 10.3390/jcm15135180 (PMC13363095; doi:10.3390/jcm15135180)
Supplement: Supplementary file 1 [file jcm-15-05180-s001.zip › jcm-4334383-supplementary.pdf]

Electronic supplement to

Hypoglossal Nerve Stimulation for Obstructive Sleep Apnea: A Systematic Review and Meta-Analysis on Responder-based Outcomes and between-study Heterogeneity

1. PICOTS question and search strategy

Table S1: PICOTS question, PICOTS formulation and derived search string

PICOTS question

Population (P)

- In adults (≥18 years) with moderate-to-severe obstructive sleep apnea diagnosed using established criteria (e.g., AHI or RDI thresholds),

Intervention (I)

- does treatment with implantable hypoglossal nerve stimulation (HNS), including unilateral or bilateral systems and breathing-synchronized, phasic, or continuous stimulation modes,

Comparator (C)

- compared with baseline (pre–post analyses), sham stimulation, standard of care (including CPAP), alternative surgical therapies, or no active comparator,

Outcomes (O)

- result in improvements in treatment response and disease severity, as measured by Sher response rate (defined as AHI <20 events/hour and ≥50% reduction from baseline), changes in AHI, ODI, daytime sleepiness (ESS), and rates of device revisions, or explantations,

Technology (T)

- across different hypoglossal nerve stimulation technologies, including unilateral and bilateral systems, and breathing-synchronized, phasic, or continuous stimulation modalities,

Study design (S)

- as reported in randomized controlled trials, non-randomized comparative studies, prospective or retrospective observational cohorts, and registry-based studies (including single-arm studies with ≥20 patients)?

PICOTS formulation

In adults with obstructive sleep apnea, what is the effectiveness and safety of implantable hypoglossal nerve stimulation, compared with baseline or alternative therapies, in improving polysomnographic outcomes, patient-reported symptoms, quality of life, and adverse event profiles across different hypoglossal nerve stimulation technologies (including unilateral and bilateral systems and different stimulation modalities), as reported in clinical trials and observational studies?

PICOTS-derived search string

```
(
  "Sleep Apnea, Obstructive"[Mesh]
  OR obstructive sleep apnea[tiab]
  OR obstructive sleep apnoea[tiab]
  OR OSA[tiab]
)
AND
(
  HNS[tiab]
```

OR "hypoglossal nerve stimulation"[tiab]
OR (hypoglossal[tiab] AND (stimul*[tiab] OR neuromodulat*[tiab] OR implant*[tiab]))
OR "upper airway stimulation"[tiab]
OR (("upper airway"[tiab] OR airway[tiab] OR tongue[tiab])
AND (stimul*[tiab] OR neuromodulat*[tiab])
AND implant*[tiab])
OR "genioglossus"[tiab] AND (stimul*[tiab] OR neuromodulat*[tiab] OR implant*[tiab])
OR Inspire[tiab]
OR "Inspire Medical"[tiab]
OR "STAR trial"[tiab]
OR Genio[tiab]
OR Nyxoah[tiab]
OR aura6000[tiab]
OR ImThera[tiab]
OR Apnex[tiab]
OR "Apnex Medical"[tiab]
)

2. Risk of bias assessment

Table S2: Risk of bias assessment

**Study ID**	**Selection / inclusion bias**		**Confounding / baseline comparability**		**Intervention classification / fidelity**		**Missing outcome data / attrition**		**Outcome measurement bias**		**Selective reporting**		**Overall risk of bias**	
	Judgement	Comments	Judgement	Comments	Judgement	Comments	Judgement	Comments	Judgement	Comments	Judgement	Comments	Judgement	Comments
Woodson 2025	high	prospective, MC single-arm study, selected population with strict eligibility criteria	high	No control group; pre-post single-arm design	low	standardized device and protocol, fixed therapeutic settings, central / blinded DISE reviewer	unclear	12M PSG completed per protocol in 88/115 participants, multiple withdrawals + loss to FU, worst-case imputation for co-primary endpoints	low	12-month full-night PSG at fixed settings with predefined validity criteria; hypopnea definition prespecified; responder definitions prespecified; PSG scoring described incl. 4% AHI/ODI; validated instruments but open-label single-arm; variable completion across questionnaires; systematic AE/SAE capture using ISO 14155:2020; independent clinical events committee + DSMB;	low	trial registered; co-primary endpoints and performance goal prespecified; analysis plan described incl. worst-case imputation and hierarchical testing	high	

										SAEs tabulated with attribution				
Han 2025	high	retrospective institutional cohort + registry cohort; large exclusions due to missing BMI/AHI data	high	non-randomized observational cohorts; BMI change groups not adjusted for confounders beyond univariable models	unclear	HGNS standard of care; heterogeneous postoperative AHI sources: PSG, PSG, HSAT; registry variability	high	169/391 institutional patients excluded; 3001/4950 ADHERE excluded; heterogeneous follow-up timing	unclear	mixed sleep study modalities; final AHI timing variable; registry timing not standardized; ESS reported; no blinding; retrospective extraction; no structured adverse event reporting in manuscript	unclear	primary BMI/AHI outcomes reported; registry-derived data; incomplete standardization across cohorts	high	
Lenze 2025	high	retrospective single-center cohort; inclusion limited to patients with >9 months available SleepSync data; patients lost earlier excluded	low	no control group; exploratory clustering without external validation; limited power; potential unmeasured confounders such as stimulation discomfort, social support	low	uniform Inspire device; standardized activation and titration pathway described; device settings and follow-up protocol clearly reported	high	exclusion of patients without 9-month data; potential overestimation of adherence; incomplete postoperative sleep study/ESS availability	low	postoperative AHI available only in subset; mix of titration and non-titration studies; adherence objectively captured via device telemetry; ESS reported in subset; retrospective extraction; open-label design; no blinding; no signal of selective complication reporting; registry-type data extraction	unclear	primary adherence outcomes predefined in methods; retrospective design; no registered protocol referenced	high	
Kaffenberger 2025	high	retrospective single-center cohort; 61 included from 379 implanted patients; inclusion required complete titration and follow-up data	unclear	within-patient comparison of titration AHI vs single-amplitude AHI; no external comparator; limited adjustment for covariates	low	stimulation protocol and titration procedures clearly described; standardized PSG titration approach	high	large proportion excluded due to incomplete data; final analytic sample represents selected subset	unclear	full in-lab PSG; direct comparison of titration vs single-amplitude within same recording; no ESS or other PRO systematically reported; no structured adverse event reporting	high	primary analytic objective reported; no protocol reference available	high	
VanLoo 2024	unclear	single-center cohort; strict FDA-aligned eligibility (AHI 15-65, BMI <32,	high	observational, single-arm pre-post; no comparator; routine-care	low	uniform Inspire system; implantation technique	low	6-month follow-up complete (39/39)	unclear	baseline: type I attended PSG; 6M follow-up AHI/ODI from mixed modalities: titration PSG, treatment AHI, and/or full-night HST; no	unclear	outcomes defined and reported at 6 months (AHI, ODI, ESS, usage,	high	single-arm observational design with high confounding;

		CPAP intolerant, no CCC on DISE); mixed prospective/retrospective registry-derived sample; predominantly male (79%)		registry setting, unmeasured confounding likely		described (2- vs 3-incision); structured post-implant activation/titration pathway described				central scoring; ESS collected, but open-label; patient-experience questionnaire has low response at 6M (18/39), response bias risk; no structured adverse-event table or systematic AE ascertainment description evident in the paper sections provided; industry funding/COI disclosed		Sher response), but no prospective protocol/registration referenced; mixed retrospective data capture increases reporting flexibility		heterogeneous follow-up sleep-study modality at 6M
Gao 2023	high	retrospective single-center chart review of HGNS patients; inclusion limited to implanted patients with available DISE and sleep study data; highly selected population meeting HGNS eligibility criteria including AHI 15–65, BMI ≤35, and absence of complete concentric collapse	high	non-randomized observational design; comparison between apnea- vs hypopnea-predominant phenotypes without adjustment for key confounders; largely univariate analyses only	unclear	all patients received HGNS implantation with DISE evaluation performed by a single otolaryngologist; standardized surgical indication and DISE assessment within one center	high	216 patients included but only 56 had 1-year postoperative AHI available for outcome assessment; large loss to follow-up acknowledged by authors and potential attrition bias discussed	unclear	postoperative AHI derived from a mix of home sleep studies (77%) and PSG (23%), introducing heterogeneity; retrospective extraction from clinical records; ESS collected pre- and post-treatment but open-label observational design; incomplete follow-up data; study focuses on phenotypic predictors of efficacy; systematic reporting of adverse events or surgical complications not clearly described	unclear	no prespecified protocol or trial registration referenced; retrospective hypothesis-driven analyses using existing dataset	high	driven mainly by retrospective single-center design and substantial attrition for the key 1-year outcome endpoint
Kent 2023	low	prospective multicenter RCT; 1:1 randomization at activation; baseline characteristics comparable between groups	low	randomized design; no major baseline imbalances in age, BMI, baseline AHI, ESS	unclear	open-label management pathways; tPSG vs home titration; ad libitum clinic visits allowed;	unclear	differential attrition: 70% completion in tPSG vs 87% in eHST; analysis largely	low	primary endpoint AHI objectively measured; 6-month outcome assessed via eHST in both groups; standardized scoring criteria applied; ESS open-label; nonblinded design; equivalence not demonstrated within predefined margin; adverse events and therapy adjustments	low	registered trial; prespecified equivalence margins; primary and secondary endpoints reported	low	

						protocol deviations possible		based on completers		described; no major safety signal imbalance				
Schwartz 2023	low	prospective multicenter RCT; 20 centers; clear eligibility criteria: AHI 20,Äì65, BMI ,â§35, PAP intolerance; randomized 2:1; baseline characteristics largely balanced; however, screened 1289, implanted 138 (10.7%), limiting external validity	low	randomized design; baseline comparability reported with standardized mean differences; minor imbalance in female proportion and baseline AHI/T90 acknowledged with sensitivity analyses	low	uniform aura6000 THN system; standardized implant and titration protocol described; prespecified activation schedule; consistent PSG scoring per AASM criteria	unclear	sample size planned for 12.5% attrition; responder analyses based on available data; no full CONSORT-style attrition breakdown in main text	low	polysomnography scored per AASM 2007 criteria; parallel-arm randomized comparison at month 4; objective AHI/ODI endpoints; bootstrapped medians shown; ESS, FOSQ, EQ-5D; open-label design, blinding not feasible; acknowledged in Limitations; potential expectancy effect; prospectively adjudicated adverse events; DSMB and clinical events committee; explicit reporting of serious/non-serious events	unclear	trial registered: NCT02263859; co-primary endpoints clearly prespecified; 1 of 4 primary endpoints not met and transparently reported; post hoc sleep-efficiency subgroup explicitly labeled as exploratory	low	rigorous multicenter randomized design with objective endpoints; open-label nature and partial reliance on responder thresholds introduce some risk, but internal validity substantially stronger than prior single-arm HNS studies
Veugen 2023	high	retrospective single-center cohort; inclusion required availability of 12-month follow-up; strict Dutch reimbursement criteria (AHI 30,Äì50, BMI <32, no CCC); highly selected CPAP-intolerant population; n=25; 96% male	high	single-arm pre-post design; no comparator group; no multivariable adjustment; improvements assessed against baseline onl	low	uniform Inspire UAS system; standardized implantation; all follow-ups performed with in-lab PSG at 2, 6, 12 months; AASM scoring described	unclear	25 included; manuscript implies complete 12-month data required for inclusion; no explicit flow diagram; unclear if any patients excluded after implantation; denominator consistency not fully	unclear	all outcomes based on full in-lab PSG; manual AASM scoring; consistent measurement modality across timepoints; ESS prospectively collected but open-label; PET questionnaire subjective and non-validated; small sample amplifies response bias; AEs systematically tabulated at 6 and 12 months; severity classified; however retrospective collection and single-center reporting; no independent adjudication	unclear	no trial registration; outcomes appear consistent with objectives	high	retrospective single-arm design, small sample size, highly selective reimbursement-driven eligibility criteria; strong internal measurement quality but high risk of confounding and limited generalizability

								transparent							
Boroosan 2022	low	Retrospective chart review/retrospective cohort at single tertiary center including consecutive implanted patients. Cohort reflects standard-of-care implanted candidates (already selected for HGNS); representativeness beyond this setting is uncertain.	high	Single-arm pre-post surgical cohort with no comparator; analyses focus on predictors with limited adjustment in small sample (n=39)	low	Intervention is UAS (Inspire system) within a real-world multicenter registry; implantation details referenced to prior publications and practice variation across >50 centers is possible.	low	One of 40 implanted patients excluded due to incomplete postoperative PSG. Some variables have missing follow-up values (e.g., postoperative oxygen saturation measures reported for fewer patients).	low	AHI measured by PSG, but when full-night postoperative PSG unavailable, AHI was taken from titration study, introducing potential non-uniform ascertainment.	unclear	Outcomes and predictor variables described are reported (AHI, Sher success). Safety outcomes are absent, and postoperative AHI source may vary (full-night PSG vs titration) without reporting how often each occurred.	high	High risk driven by confounding (single-arm design) and lack of safety reporting; additional concerns from variable outcome ascertainment (titration AHI when PSG unavailable).
Heiser 2022	high	first 10 historical uniHNS cases from 2014 vs first 10 biHNS cases from 2020; non-concurrent cohorts; small sample; one dropout in biHNS)	high	non-randomized observational comparison; temporal separation; no matching/propensity adjustment; small n	unclear	different activation timelines; uniHNS month-2 titration + month-3 PSG; biHNS month-3 titration PSG; different stimulation paradigms	unclear	one dropout in biHNS; ESS available only for subsets at month 3; small numbers amplify impact	unclear	month-3 PSG for both; however biHNS month-3 PSG is titration PSG; uniHNS month-3 PSG follows prior titration; whole-night AHI reported; ESS used; open-label; no blinding; no adverse events reported; very small sample; short 3-month follow-up	unclear	outcomes reported as defined; no protocol reference; pilot comparative design	high	
Wang 2022	high	retrospective single-center review; inclusion required available pre- and postoperative sleep studies;	high	single-arm pre-post design; no comparator; responder vs nonresponder subgroup analysis	low	uniform Inspire HGNS system; standardized implantation with detailed	unclear	46 included with both pre- and postoperative data; no explicit flow diagram;	unclear	pre- and postoperative AHI derived from mixed modalities (PSG and HSAT); modality heterogeneity may affect comparability; no central scoring reported; no ESS collected pre- and postoperatively; open-label; no	unclear	primary aim neurophysiological; clinical efficacy outcomes reported but no registered protocol; multiple	high	retrospective design, small sample, mixed sleep-study modalities, no comparator, exploratory subgroup

		n=46; mixed PSG and HSAT modalities; predominantly male (92%); strict FDA-like inclusion criteria (AHI 15-65, BMI <35, no CCC)		observational; no multivariable adjustment beyond BMI comparison		intraoperative NIM protocol; activation and titration pathway described		unclear if any implanted patients excluded due to missing follow-up; postoperative window 6-12 months variable		blinding; timing within 6-12 month window variable; no structured adverse-event reporting; safety outcomes not systematically presented; focus on neurophysiology rather than clinical safety		subgroup analyses (responders vs nonresponders) without correction for multiplicity		analyses; strong intraoperative measurement fidelity but limited internal validity for clinical effectiveness inference
Suurna 2021	high	prospective registry; participation voluntary; 1,849 enrolled but only 823 completed final visit and 782 had outcome data; large loss between enrollment and analyzable cohort; predominantly Caucasian (95%) and male (73%), limited representativeness	high	observational registry without control group; pre-post comparisons only; BMI subgroup analyses non-randomized; discomfort analyses observational; no multivariable adjustment reported in primary analyses	unclear	standard UAS system and routine-care management described; however, multicenter real-world setting with variability in PSG vs HSAT, follow-up timing (up to 2 years), and therapy optimization practices	high	1,019 eligible for final visit, 823 completed, 782 with reported outcomes; substantial missing AHI/ESS data; authors acknowledge incomplete follow-up; multiple imputation performed but primary results based on completers	unclear	final AHI based on full-night PSG or HSAT, but modality not standardized across sites; no central scoring; heterogeneity in diagnostic equipment; registry-level data collection; ESS prospectively collected but open-label; satisfaction and CGI-I subjective; no blinding; potential expectancy bias; large-scale registry capture; explicit SAE reporting (2.3%); however, site-reported events without independent adjudication; sponsor-supported registry	unclear	registry registered; predefined outcomes; multiple subgroup and exploratory analyses (BMI, discomfort); potential reporting flexibility; industry funding and honoraria disclosed	high	large real-world prospective registry improves external validity, but high attrition, lack of comparator, observational subgroup analyses, and heterogeneity in measurement introduce substantial risk of bias for effectiveness inference
Flynn 2021	high	retrospective cohort study from a single tertiary-care center including only patients who underwent UAS implantation between	high	non-randomized observational design; comparison between responders and non-responders without multivariable	unclear	all patients received the same Inspire upper airway stimulation device with standardized preoperative	unclear	95 patients included but only 83 had both pre- and post-intervention PSG data used for outcome	low	post-intervention AHI derived from titration polysomnography rather than standardized follow-up PSG; estimated treatment AHI used due to variable titration conditions; ESS used pre- and post-treatment; open-label surgical cohort with no blinding and potential reporting	unclear	retrospective study without clearly referenced prespecified protocol or trial registration; analyses appear exploratory	high	driven by retrospective single-center design, responder-based subgroup analysis, and limited control for

		2016–2019; eligibility restricted to PAP-intolerant patients with BMI <35 and without complete concentric velopharyngeal collapse on DISE		adjustment; potential confounding from patient selection and clinical characteristics		workup including PSG and DISE; activation and titration protocol described		analyses, suggesting moderate missing outcome data though attrition reasons are not fully detailed		bias; study focuses on efficacy predictors; systematic safety or complication reporting is not a primary endpoint and not comprehensively detailed				confounding factors
Patil 2020	high	retrospective case series of 53 consecutive veterans undergoing HNS implantation at a single VA academic center; population restricted to PAP-intolerant patients referred for surgery, limiting external validity	unclear	non-randomized observational design comparing COMISA vs OSA-only groups with substantial baseline differences, e.g., higher rates of PTSD, depression, and anxiety in the COMISA group; no multivariable adjustment performed	low	all patients received the same Inspire HNS system, implanted by a single surgeon, with standardized activation and titration protocols	low	moderate missing data across several outcomes: 3 patients without postoperative PSG, 8 without ISI, 4 without PCL-5, and 2 without adherence checks; reasons partly reported but attrition remains non-negligible	low	postoperative efficacy assessed using titration PSG rather than standardized follow-up full-night PSG; some patients used home sleep testing; this may introduce measurement variability; ESS and ISI used but study is open-label without blinding, and insomnia severity was mainly assessed postoperatively, introducing potential recall and reporting bias; adverse events were reported but safety was not a primary endpoint, and systematic prospective monitoring is not clearly described	unclear	retrospective design with no prespecified protocol or trial registration; analyses appear exploratory	low	driven by retrospective single-center design, small sample size, heterogeneous baseline characteristics, and moderate missing outcome data
Huntley 2021	high	retrospective international multicenter cohort; non-concurrent groups; only patients with follow-up sleep study included	high	non-randomized case-control design; baseline age and BMI significantly different; no propensity matching or	unclear	heterogeneous traditional surgeries; follow-up timing differs significantly; mixed PSG/HST modalities; sleep	high	traditional surgery: 233 baseline, 196 final AHI; ESS available only for subsets; different follow-up durations	high	traditional surgery sleep study type not available; UAS 70% PSG / 28% HST; inconsistent scoring rules acknowledged by authors; ESS used; retrospective extraction; incomplete data capture; no safety outcomes systematically collected or reported for either cohort	high	primary outcomes reported; retrospective design; heterogeneous data sources	high	

				multivariable adjustment		study scoring rules not standardized across surgery cohort		between groups						
Patil 2021	high	retrospective single-center case series of 53 consecutive veterans undergoing HNS implantation; highly selected PAP-intolerant population	high	non-randomized comparison of COMISA vs OSA-only groups with major differences in psychiatric comorbidities; no multivariable adjustment	low	all patients received Inspire HNS implanted by a single surgeon with standardized candidacy evaluation including DISE	unclear	53 implanted; 3 without postoperative PSG, additional missing PROs such as ISI and PCL-5	unclear	postoperative titration PSG used to determine treatment AHI, not independent follow-up PSG; insomnia severity assessed only postoperatively, introducing recall bias; adverse events explicitly reported, including pneumothorax, device issues, and infection	unclear	retrospective study without prespecified protocol	high	retrospective design, selected veteran population, and partial outcome data
Chao 2021	high	retrospective chart review of 68 patients from a single tertiary-care center undergoing HGNS implantation	low	non-randomized design comparing success vs failure groups without multivariable adjustment	low	all patients received the Inspire HGNS system, implanted by a single surgeon with consistent evaluation including DISE	unclear	postoperative outcomes sometimes derived from titration PSG rather than standardized follow-up PSG, with incomplete formal follow-up studies	high	postoperative AHI often based on titration studies or mixed modalities, which may overestimate success; primary outcomes are objective PSG metrics rather than PROs; safety reporting not a primary endpoint and not systematically described	unclear	retrospective exploratory analysis without prespecified protocol	high	retrospective single-center observational design
Patel 2020	unclear	retrospective single-center cohort; inclusion limited to implanted patients with available pre- and post-PSG; standard	high	no untreated control group; effectiveness assessed pre-post; subgroup comparisons observational; profound OSA	low	uniform Inspire device; implantation by two senior surgeons; standardized activation and 6,Äì8	unclear	analysis restricted to patients with both pre- and post-implant PSG; ESS incompletely available	unclear	post-op AHI derived from titration PSG, not full-night independent PSG; treatment AHI defined at maximally tolerated voltage, potential context-related measurement bias, ESS collected retrospectively; incomplete availability in profound subgroup; open-label design;	unclear	primary outcome defined; regression analysis reported; no registered protocol referenced; exploratory subgroup	high	retrospective non-comparative design, subgroup analyses with small n, titration-based outcome assessment,

		UAS candidacy incl. DISE-based CCC exclusion, limited generalizability		subgroup very small n=9, limited power, residual confounding likely		week titration PSG protocol described		in profound subgroup; no detailed attrition flow diagram		no blinding, no structured adverse event reporting described; focus primarily on efficacy; conflicts of interest disclosed		analyses increase multiplicity risk		residual confounding
Vonk 2020	high	retrospective single-center cohort; 47 implanted, 44 analyzed (exclusion of 3 without titration data); strict Dutch reimbursement criteria (AHI 30-50 for reimbursement period; BMI <32; no CCC); highly selected CPAP-intolerant population; predominantly male (86%)	high	single-arm pre-post design; no comparator group; no adjustment for confounders; subgroup DISE analysis exploratory and underpowered	unclear	uniform Inspire system; standardized implantation/activation; however outcomes based on post-titration PSG under therapeutic settings (treatment AHI), not full-night habitual setting, optimized efficacy measurement	unclear	3/47 excluded due to lack of titration data; final n=44; no long-term follow-up; unclear handling of incomplete titration nights; denominator consistency acceptable but no flow diagram	unclear	postoperative AHI derived from titration PSG using therapeutic window rather than whole-night real-world setting; may overestimate effect; same modality pre/post; no validated subjective outcomes (e.g., ESS) reported due to lack of data; absence of PROs limits assessment of symptomatic benefit; therapy-related AEs systematically tabulated (Table 4); retrospective reporting; high rate of stimulation-related discomfort (45%); no independent adjudication	unclear	primary outcomes aligned with objectives; no trial registration; subgroup DISE analysis post hoc; reliance on treatment AHI not prespecified as superiority measure	high	retrospective design, no comparator, optimized titration-based outcome assessment, short-term follow-up, selective highly screened population; strong internal PSG measurement but limited external validity and high confounding risk
Sarber 2020[1]	high	retrospective single-center salvage cohort; inclusion restricted to patients intentionally implanted outside FDA criteria; highly selected population; small sample n=18; predominantly	high	no control group; purely pre-post comparison; heterogeneous off-label indications, AHI <15, AHI >65, BMI >32, CAI >25%, some outside criteria; no multivariable adjustment;	low	uniform Inspire device; single surgeon; standardized DISE screening, activation and titration protocol described; consistent postoperative titration PSG	unclear	small cohort; adherence reported at first timepoint only; postoperative outcomes based on titration PSG; no detailed loss-to-follow-up diagram	unclear	postoperative AHI derived from titration PSG at optimal voltage rather than independent full-night PSG; authors acknowledge likely overestimation of efficacy; ESS collected retrospectively at clinic follow-up; open-label design; no blinding; timing variable; perioperative complications explicitly described; minor event rate reported; retrospective capture may miss minor adverse events	unclear	primary endpoints stated; nonparametric analysis used; no registered protocol; exploratory subgroup reporting without correction for multiplicity	high	retrospective off-label salvage cohort, small n, no comparator, heterogeneous indication groups, titration-based outcome assessment

		older white male veterans ,Üí limited generalizability		very small subgroups		methodology								
Eastwood 2020	high	prospective single-arm feasibility study of bilateral HGNS; small sample (n≈27) and highly selected CPAP-intolerant OSA population meeting strict eligibility criteria including BMI ≤35 and absence of complete concentric collapse	high	no control group; pre–post single-arm design	low	standardized implantation of the Genio bilateral hypoglossal nerve stimulation system with defined activation and titration protocol	low	moderate attrition between implantation and final outcome assessment; not all implanted patients contributed to final PSG outcomes	low	primary outcomes assessed using full-night polysomnography at predefined follow-up; ESS and quality-of-life measures reported in an open-label single-arm design; adverse events systematically collected and reported as part of the clinical feasibility trial	low	no explicit protocol registration referenced; early feasibility study with exploratory endpoints	low	driven mainly by small sample size and single-arm design despite standardized measurements
Lee 2019	high	combined prospective + retrospective two-center cohort; inclusion limited to implanted patients with available therapeutic PAP level and full postoperative efficacy study; excludes missing sleep studies; highly selected PAP-	high	non-randomized comparison of PAP < 8 vs >8 cmH, imbalance in prior airway surgery; no multivariable adjustment for key covariates; small low-PAP subgroup n=13	low	standard HGNS system; full-night efficacy PSG at single optimized setting; adherence verified via device telemetry/Inspire Cloud when available	unclear	56 included after exclusion for missing data; ODI and questionnaire data incomplete in subsets; unclear how many total implanted during study window	unclear	postoperative outcomes based on full-night efficacy studies @ 6 months; AHI and ODI extracted from PSG/HSAT; objective definitions of response prespecified; ESS and Snoring VAS reported; open-label surgical study; no blinding; postoperative values sometimes averaged across visits; no structured adverse event reporting framework presented; safety not primary focus in results tables	unclear	primary outcomes defined a priori; sample size calculation reported; no registered protocol referenced; multiple responder definitions explored	high	

		intolerant population												
Mahmoud 2019	high	retrospective single-center implanted cohort; candidacy pathway/DISE selection limits generalizability; analysis excludes patients with incomplete follow-up/data	high	no external comparator; effectiveness largely pre-post in treated patients; residual/unmeasured confounding inherently likely in retrospective single-arm context	low	device and pathway clearly described; implantation/activation/titration workflow reported; laterality largely consistent	unclear	91 implanted, 82 analyzed; exclusions include incomplete data/lost follow-up and one explant prior to titration, potentially outcome-related missingness	unclear	post-op AHI derived from titration study rather than full-night PSG; authors note variable AHI duration, measurement context limitations, ESS/FOSQ not presented as outcomes in this paper, AEs reported with counts/rates, but capture method/time window not clearly described as systematic, potential under-ascertainment	unclear	key outcomes described and reported, but no protocol/registry referenced; titration-study endpoint choice and AE ascertainment details limit transparency	high	high confounding due to retrospective non-comparative design + some concerns on measurement/attrition
Huntley 2019	high	retrospective review; inclusion required completed postoperative PSG; UAS largely replaced TORS over time; temporal selection bias explicitly acknowledged	high	non-randomized comparison; significant baseline age difference; prior surgery differences; no multivariable adjustment	unclear	standardized definitions provided; however TORS is a multilevel surgical approach in many patients; UAS activation/titration details not fully standardized in text	high	37 TORS performed, 24 analyzed; 94 UAS performed, 76 analyzed; exclusions based on follow-up PSG	high	postoperative AHI values reported; unclear whether full-night standardized PSG or titration PSG across both groups; measurement conditions not harmonized; no ESS or other PRO reported in results tables; major complications defined a priori; readmission, LOS, and complications explicitly reported	unclear	primary outcomes reported; retrospective design; no protocol reference	high	
Steffen 2019	high	retrospective single-center cohort; inclusion restricted to patients with complete M12 and M24 follow-up; 5/30	high	non-randomized subgroup comparison (UPPP after vs before vs none); baseline AHI significantly higher in	unclear	uniform Inspire UAS implantation; however, UPPP-TE performed variably before or after	high	5/30 excluded for missing M12/M24 or reoperation; analysis restricted to complete cases; no	unclear	baseline and follow-up assessed via home sleep testing (HST), not full PSG; month-2 titration PSG only; repeated DISE only in group 1; potential measurement heterogeneity, ESS collected but retrospective design; open-label; no blinding; small	unclear	multiple responder definitions (Sher criteria, AHI <15, AHI <5); subgroup emphasis post hoc; no registered	unclear	retrospective design, small heterogeneous subgroups, confounding by indication, complete-case analysis, HST-

		excluded due to incomplete follow-up or reoperation; small total n=25; subgroup sizes very small (7/10/8); highly selected PAP-intolerant population		group 1; no multivariable adjustment; subgroup allocation based on clinical response pathway, strong confounding by indication		implantation; advanced titration and DISE-guided adjustments applied selectively; learning-curve effects acknowledged		intention-to-treat approach; small n amplifies attrition impact		subgroup sizes; no structured adverse event reporting framework; limited safety data presented; focus primarily on efficacy outcomes		protocol; exploratory subgroup comparisons		based outcomes; internal validity limited for causal inference regarding UPPP timing effects
Huntley 2018	high	retrospective review; inclusion required completed postoperative sleep study; elevated-BMI subgroup offered UAS based on clinical assessment	high	non-randomized case-control comparison BMI>32 vs <32; no adjustment beyond univariate tests; baseline AHI trend higher in BMI>32	low	standardized operative details and postoperative management described; activation + titration PSG protocol specified	high	excluded any patient without postoperative sleep study; extent of exclusions not fully quantifiable from the manuscrip	high	postoperative outcomes taken from titration PSG/ optimal titration AHI, not full-night follow-up PSG/HST; ESS reported; retrospective extraction; no blinding; no systematic adverse event reporting	unclear	outcomes reported as described; retrospective design; no protocol reference	high	
Zhu 2018	high	non-randomized matched cohort; consecutive ‚â•65y patients (n=31) matched to younger controls (n=31) by AHI/BMI; no random allocation; potential residual confounding; no BMI upper limit; small sample	unclear	matching performed for AHI and BMI; groups similar for ESS and ODI; however imbalance in sex distribution and comorbidity burden (higher Charlson index in older group); no multivariable adjustment	low	uniform Inspire II system; standardized 3-incision implantation; defined activation and PSG titration protocol; same scoring criteria; identical follow-up schedule at 6 and 12 months	unclear	baseline n=31 per group; follow-up n decreases (e.g., M12: study n=25, control n=20); no CONSORT-style flow diagram; reasons for attrition not clearly detailed; denominators vary	unclear	baseline and M2 titration via PSG; 6- and 12-month follow-up via home sleep testing (HST); modality shift may affect comparability; Sher criteria applied post hoc; ESS collected prospectively; open-label design; no blinding; no validated QoL instrument beyond ESS; states no serious perioperative complications; adverse events described narratively; no structured AE table; short 12-month horizon	unclear	primary endpoint defined as AHI <15; Sher criteria additionally applied; no protocol registration; limited transparency on multiplicity	high	small, non-randomized matched cohort; short-term follow-up; mixed sleep-study modalities; no adjustment for confounding; internal consistency acceptable but external validity and causal inference limited

								across endpoints						
Woodson 2018	high	multicenter prospective cohort but highly selected population: CPAP intolerant, BMI <32, AHI 20,Äì50, no CCC on DISE; 126 implanted, 97 (78%) completed 5-year protocol, 71 (56%) underwent voluntary 5-year PSG (potential selection bias)	high	single-arm design; no concurrent control; long-term comparisons vs baseline only; post-hoc responder analyses; industry-sponsored	low	uniform Inspire system; standardized implantation; independent core-lab PSG scoring; predefined responder definition (50% reduction & AHI <20); structured follow-up	high	29/126 did not complete 5-year follow-up; only 71/126 had 5-year PSG; deaths/explants counted as nonresponders in LOCF; multiple imputation and LOCF performed but missingness substantial and non-random (higher baseline AHI/ODI in non-completers)		in-lab PSG; dual core-lab scoring; standardized AASM criteria; responder definition predefined; sensitivity analyses performed; ESS and FOSQ prospectively collected but open-label; no blinding; placebo effect possible; however sustained effect over 5 years; systematic AE reporting; serious device-related events clearly quantified (6%)	unclear	protocol-driven trial with predefined endpoints; multiple sensitivity analyses reported; however voluntary PSG subset and responder analyses create analytical flexibility	high	prospective design and strong measurement fidelity, but absence of control group and substantial 5-year attrition with voluntary PSG subset materially increase risk of bias in long-term effectiveness estimates
Huntley 2018	high	retrospective review; inclusion required completed postoperative sleep study; large exclusion of ESP patients without follow-up PSG	high	non-randomized comparison; significant baseline differences in age, gender, and preoperative AHI	unclear	heterogeneous surgical techniques in ESP group; UAS outcomes based on titration PSG	high	97 ESP -> 33 included; 96 UAS -> 75 included; exclusion of patients without postoperative PSG	unclear	UAS outcomes derived from titration PSG; sleep study type not standardized between groups; ESS used; retrospective design; no blinding	unclear	primary outcomes reported; retrospective design; differential measurement methods	high	
Mahmoud 2018	high	retrospective single-center chart review; inclusion limited to patients who returned for postoperative PSG; highly selected CPAP-	high	non-randomized comparison of prior surgery vs no prior surgery; small comparator group (n=17); no multivariable adjustment	low	uniform implantation by single surgeon; standardized activation and titration protocol clearly described	unclear	49 implanted, 47 analyzed; 2 did not return for PSG; unclear if any additional missing ESS data	unclear	postoperative AHI derived from titration PSG at 1 month, not independent follow-up PSG; ESS reported; open-label design; no blinding; complications systematically recorded; no major adverse events; perioperative outcomes clearly described	unclear	primary endpoints defined; retrospective design; no protocol reference	high	

		intolerant population												
Shah 2018	high	retrospective case series; HNS cohort n=20 vs historical UPPP cohort n=20 selected from 116; non-contemporaneous groups (2003,Äì2012 vs 2015,Äì2016); DISE used only in HNS group; baseline age markedly different (42 vs 62 years); highly selected CPAP-intolerant patients	high	non-randomized historical comparison; significant age imbalance; heterogeneous UPPP procedures incl. adjunctive surgeries; no multivariable adjustment; purely pre-post within-group analyses with between-group comparison descriptive	unclear	HNS standardized per STAR criteria and single technique; UPPP heterogeneous techniques ¬± additional procedures; variability in surgical exposure and perioperative care	unclear	ESS available only in subsets; postoperative PSG timing variable (UPPP 60,Äì395 days); no flow diagram; unclear loss-to-follow-up handling	unclear	postoperative PSG performed, but timing differed substantially between groups; UPPP follow-up window broad; no central scoring; paired t-test approach only); ESS retrospectively extracted; incomplete availability; open-label; no blinding; small sample; no structured adverse event reporting; safety comparison not systematically presented; limited transparency beyond narrative statements	unclear	primary endpoint not explicitly prespecified; success defined post hoc using Sher criteria; no protocol registration; historical cohort selection introduces reporting flexibility	high	retrospective non-contemporaneous comparison, small n, major baseline imbalance, heterogeneous interventions, no adjustment for confounders; internal validity weak for comparative effectiveness inference
Steffen 2018	unclear	prospective multicenter single-arm study; consecutive patients; strict inclusion criteria (AHI 15,Äì65, BMI ,â§35, DISE exclusion of CCC); highly selected CPAP-intolerant population, limited generalizability	high	no control group; purely pre-post comparison; single-arm effectiveness evaluation; no adjustment for potential confounders	low	uniform Inspire UAS system; standardized implantation, activation, and titration pathway; multicenter but technique described and aligned with STAR protocol	unclear	60 enrolled; 56 completed 12-month HST; 4 did not complete follow-up (1 explant, 3 lost); responder analysis presented both completer and ITT-style including nonresponders	unclear	baseline and follow-up outcomes based on 2-night home sleep testing (HST), not in-lab PSG; HST may underestimate AHI; paired t-test comparison; no blinded central scoring described; ESS and FOSQ prospectively collected but open-label single-arm design; no blinding; potential expectancy effect; prospectively reported adverse events; only 1 explant; explicit safety section; device usage objectively captured	low	trial registered (NCT02293746); predefined outcomes; 6- and 12-month data consistently reported; responder analysis transparent	high	prospective multicenter design strengthens internal consistency, but single-arm pre-post structure without comparator results in high confounding risk for effectiveness inference

Hofauer 2017	high	single-center consecutive CPAP-intolerant cohort; BMI < 35; DISE screening; highly selected population	high	single-arm pre-post design; no comparator; no adjustment	low	standardized activation timeline; inpatient PSG titration at month 2; repeat PSG at month 3; protocol clearly described	low	no patient lost to follow-up; n=26 completed 3-month evaluation	low	inpatient 18-channel PSG according to AASM 2012 criteria; scoring rules explicitly described; ESS validated instrument; open-label design; no blinding	unclear	outcomes defined and reported; no protocol comparison; single-center observational study	high	
Huntley 2017	high	retrospective review; inclusion required completed postoperative titration PSG; patients without adequate follow-up excluded	high	single-arm cohort comparison between institutions; no control group; no multivariable adjustment	low	standardized perioperative algorithm described; uniform activation and titration protocol at both centers	unclear	only patients completing postoperative PSG included; unclear proportion of total implanted patients excluded	high	postoperative AHI derived from titration PSG at optimal stimulation parameters	unclear	primary outcomes reported; retrospective design; no prespecified protocol reference	high	
Heiser 2017	high	consecutive single-center cohort; CPAP-intolerant; BMI <35; DISE screening; highly selected population	high	single-arm prospective study; no control group; no adjustment	low	implantation protocol clearly described; standardized titration PSG at months 2 and 3; structured follow-up schedule	low	no patient lost to follow-up; all 31 completed 12 months	low	mixed measurement methods: in-lab PSG at M2/M3; home polygraphy at M6/M12; ESS validated instrument; open-label design; no blinding; adverse events defined; recorded throughout observation; serious AEs explicitly reported as absent	unclear	outcomes defined and reported; no protocol reference; single-center routine study	high	
Friedman 2016	high	prospective multicenter single-arm feasibility study; highly selected CPAP-intolerant OSA patients; 93	high	no control group; pre–post design only	low	standardized implantation of the ImThera aura6000 targeted hypoglossal	unclear	46 implanted; 43 completed 6-month PSG follow-up	low	primary outcomes AHI and ODI measured using in-laboratory PSG at baseline and 6 months; ESS and SAQLI measured in open-label single-arm design; adverse events systematically recorded and classified as serious/non-	unclear	registered trial, but exploratory predictor analyses performed post-hoc	high	mainly due to single-arm feasibility design and highly selected patient population

		screened → 46 implanted				neurostimulation system with defined activation and titration protocol				serious with procedural attribution				
Kezirian 2014	high	prospective single-arm trial; highly selected PAP-intolerant patients; BMI limits; exclusion of prior airway surgery	high	no control group; pre-post single-arm design	low	implantation protocol clearly described; standardized titration PSG; central core lab scoring; uniform activation algorithm	unclear	31 activated; 3 missing 12-month data; last observation carried forward used; 4 total explants including 1 infection	low	Full in-lab PSG; central core laboratory scoring; predefined AASM criteria; validated instruments used (ESS, FOSQ, SAQLI, PSQI, BDI); open-label design; no blinding; systematic AE reporting; independent Clinical Events Committee; Kaplan-Meier time-to-event analysis	low	predefined primary endpoints (AHI, FOSQ); trial registration (NCT01186926 / NCT01211444); outcomes reported at 6 and 12 months	high	
Van de Heyning 2012	high	prospective multicenter but highly selective feasibility cohort; CPAP-intolerant moderate-severe OSA; part 1 broad criteria then post hoc refinement; only 31/61 enrolled received implant; exclusion of 3 subjects from analysis who did not reach 6 months (Fig. 2); highly selected population	high	nonrandomized, single-arm pre-post design; part 2 selection derived from part 1 response predictors (AHI ,â§50, BMI ,â§32, no CCC); electrode placement differed between parts; strong confounding by indication and protocol modification	unclear	same Inspire II UAS platform; however electrode placement differed between part 1 (main trunk) and part 2 (medial branch only); titration protocol standardized but iterative adjustments performed	high	61 enrolled -> implanted -> 28 analyzed; 3 excluded from 6-month analysis; complete-case analysis only; no ITT; small n amplifies impact of attrition	unclear	full in-lab PSG per AASM guidelines pre- and post-implant (Methods p.3); prespecified responder definition; paired statistical testing; however open-label and small subgroups; ESS and FOSQ prospectively collected; open-label; no blinding; potential expectancy bias; serious and non-serious AEs reported descriptively (pp.5,Äì6); sponsor-funded study; short 6-month follow-up; no independent adjudication described	unclear	primary endpoint defined (AHI change at 6 months); responder criteria prespecified; however part 2 derived from part 1 predictors; post hoc subgroup analyses (responders vs nonresponders); no trial registration reported	high	feasibility, small sample, protocol modification mid-study, complete-case analysis, no comparator; internal validity limited though objective PSG endpoints strengthen measurement reliability

[1] Data extracted from the off-label subgroup (patients implanted outside U.S. FDA eligibility criteria; n = 18), which is the primary analysis population of this publication. Two patients were excluded from postoperative assessment (1 device explantation due to infection, 1 refusal of titration PSG), yielding 16 evaluable patients (13 Sher responders) as reported in Figure 2.

3. Forest plots of effect estimates of secondary outcomes

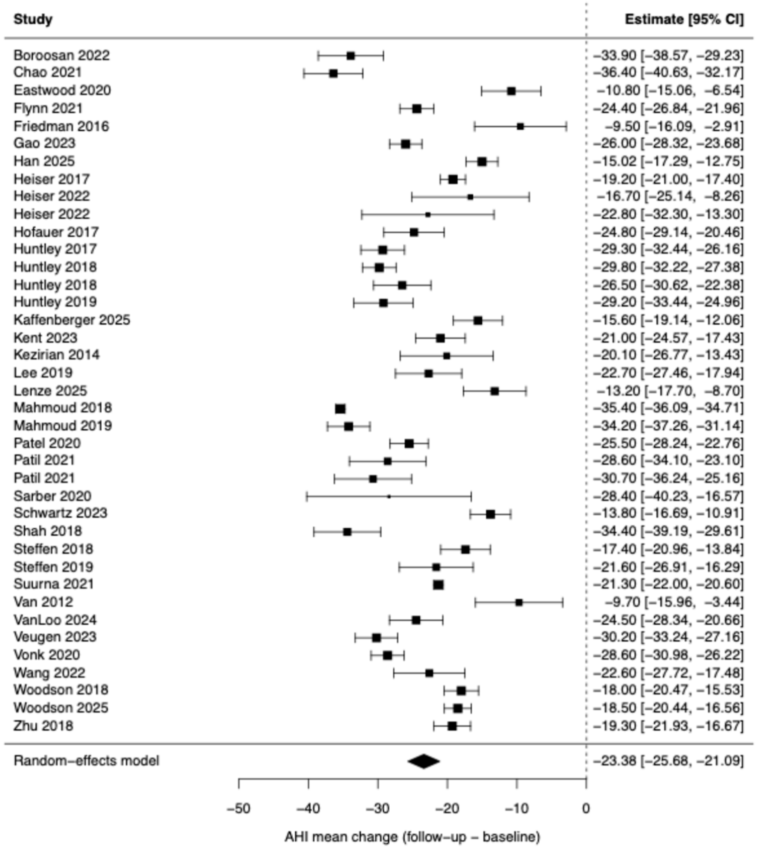

Figure S1: Forest plot of AHI changes (pre-post analysis)

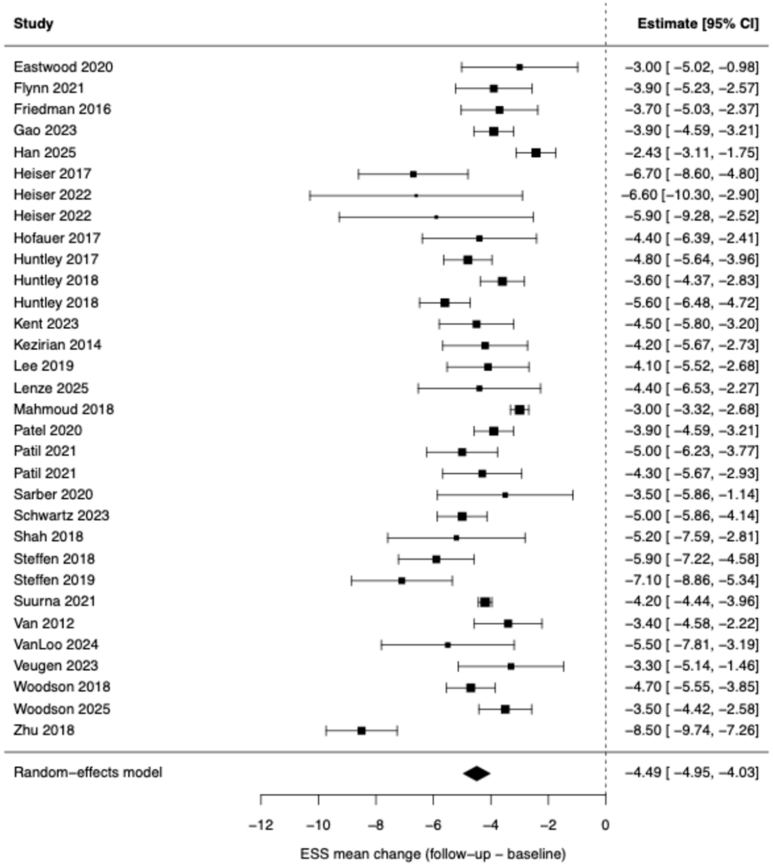

Figure S2: Forest plot of ESS changes (pre-post analysis)

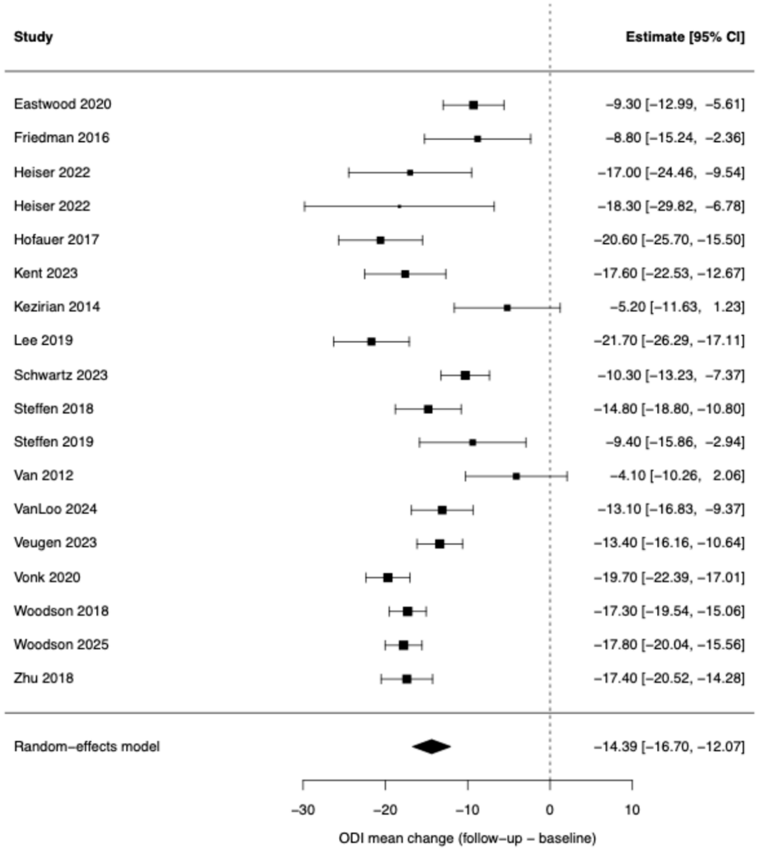

Figure S3: Forest plot of ODI changes (pre-post analysis)

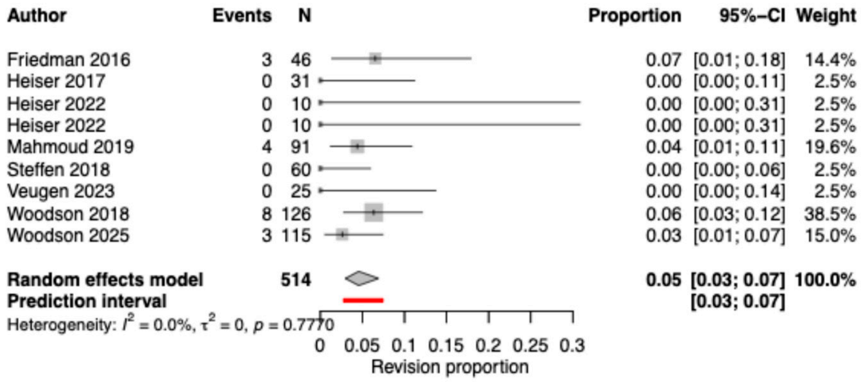

Figure S4: Forest plot of revision rates

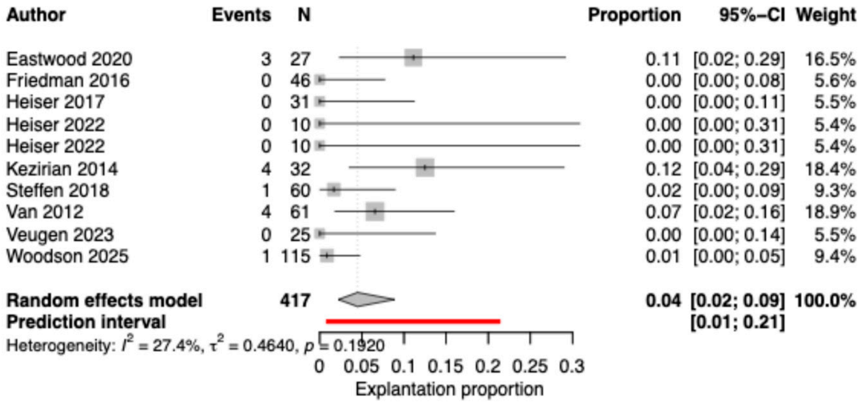

Figure S5: Forest plot of explantation rates

4. Eggers test for assessment of publication bias

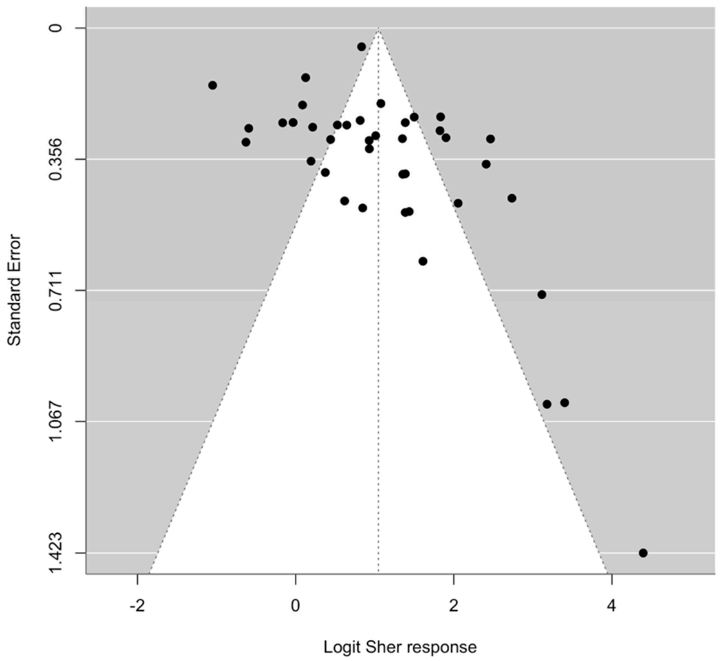

Figure S6: Eggers funnel plot for outcome Sher response

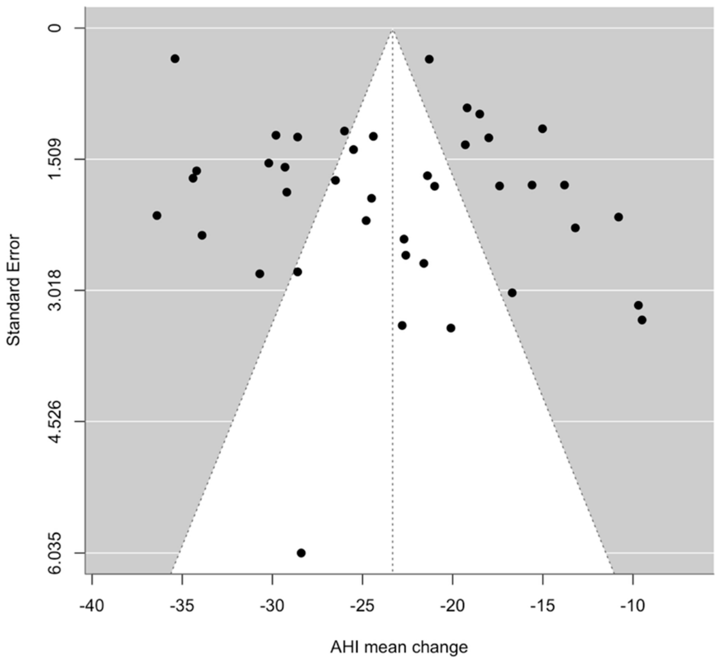

Figure S7: Eggers funnel plot for outcome AHI change

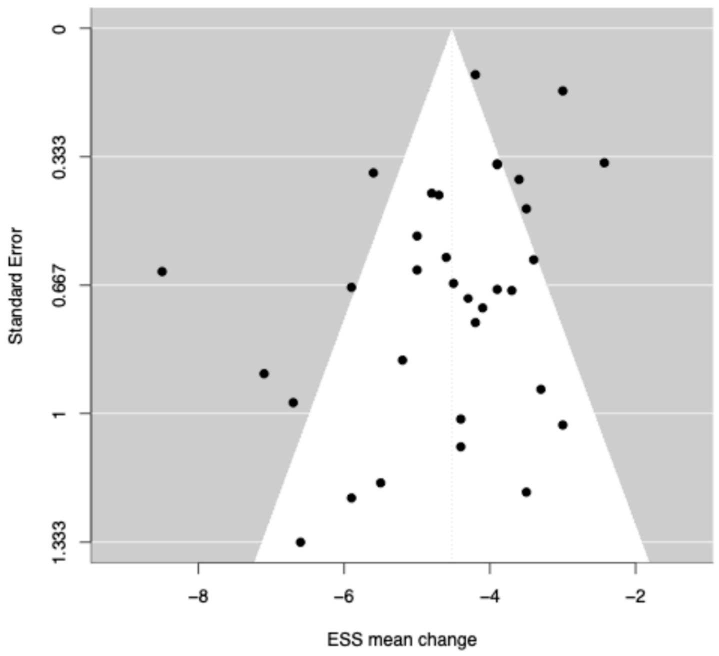

Figure S8: Eggers funnel plot for outcome ESS change

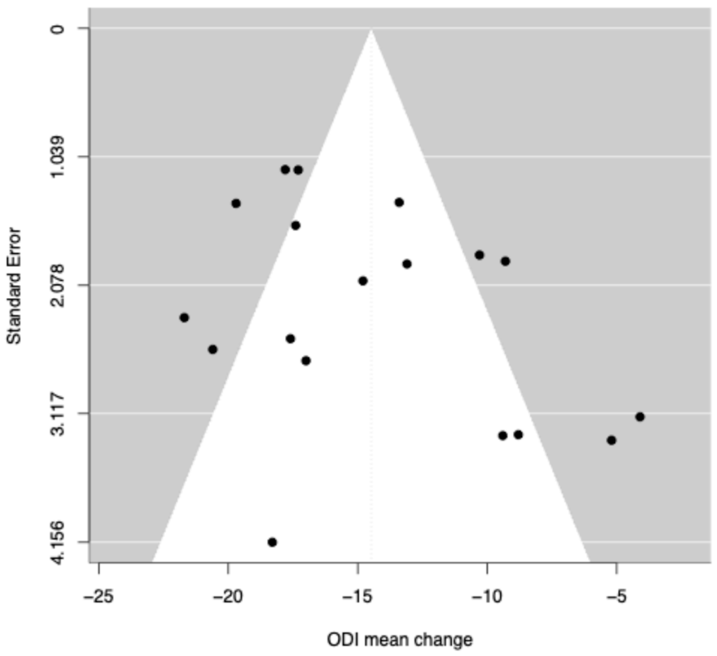

Figure S9: Eggers funnel plot for outcome ODI change

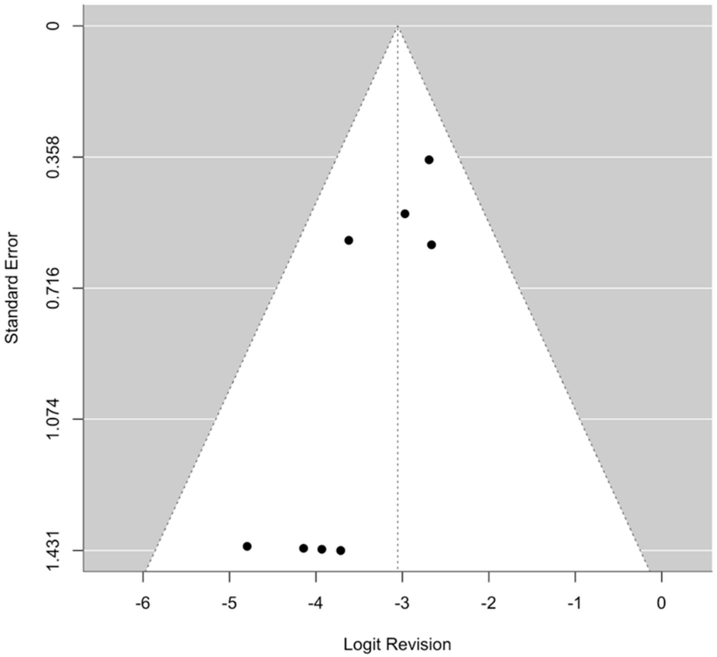

Figure S10: Eggers funnel plot for outcome Revision rate

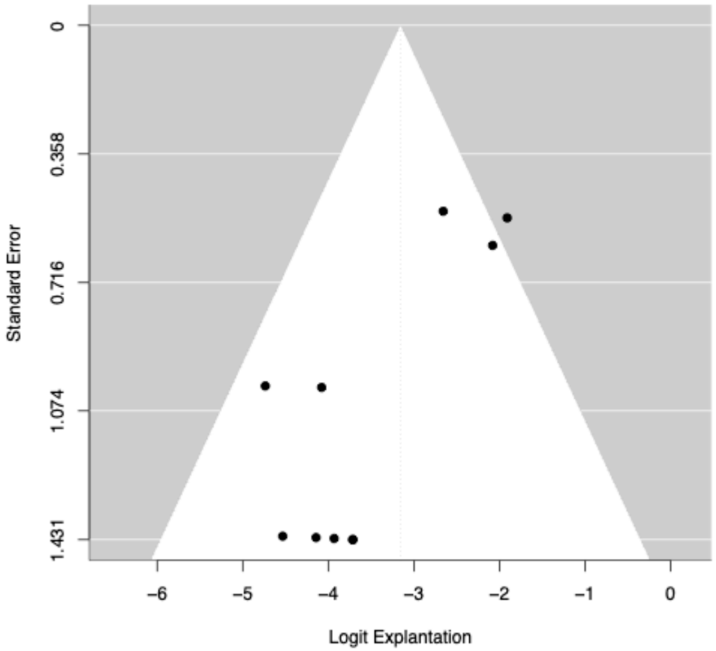

Figure S11: Eggers funnel plot for outcome Explantation rate
